# Co-circulation of *Orthobunyaviruses* and Rift Valley Fever Virus in Mauritania, 2015

**DOI:** 10.3389/fmicb.2021.766977

**Published:** 2021-12-24

**Authors:** Nicole Cichon, Yahya Barry, Franziska Stoek, Abdellah Diambar, Aliou Ba, Ute Ziegler, Melanie Rissmann, Jana Schulz, Mohamed L. Haki, Dirk Höper, Baba A. Doumbia, Mohamed Y. Bah, Martin H. Groschup, Martin Eiden

**Affiliations:** ^1^Institute of Novel and Emerging Infectious Diseases, Friedrich-Loeffler-Institut, Greifswald-Insel Riems, Germany; ^2^Office National de Recherche et de Développement de l’Elevage (ONARDEL), Nouakchott, Mauritania; ^3^Department of Viroscience, Erasmus Medical Center, Rotterdam, Netherlands; ^4^Institute of Epidemiology, Friedrich-Loeffler-Institut, Greifswald-Insel Riems, Germany; ^5^Institute of Diagnostic Virology, Friedrich-Loeffler-Institut, Greifswald-Insel Riems, Germany; ^6^Ministère du Développement Rural, Nouakchott, Mauritania

**Keywords:** NRIV, BUNV, BATV, RVFV, Mauritania, co-circulation

## Abstract

Ngari virus (NRIV) has been mostly detected during concurrent outbreaks of Rift Valley fever virus (RVFV). NRIV is grouped in the genus *Orthobunyavirus* within the *Bunyaviridae* family and RVFV in the genus *Phlebovirus* in the family *Phenuiviridae*. Both are zoonotic arboviruses and can induce hemorrhagic fever displaying the same clinical picture in humans and small ruminants. To investigate if NRIV and its parental viruses, Bunyamwera virus (BUNV) and Batai virus (BATV), played a role during the Mauritanian RVF outbreak in 2015/16, we analyzed serum samples of sheep and goats from central and southern regions in Mauritania by quantitative real-time RT-PCR, serum neutralization test (SNT) and ELISA. 41 of 458 samples exhibited neutralizing reactivity against NRIV, nine against BATV and three against BUNV. Moreover, complete virus genomes from BUNV could be recovered from two sheep as well as two NRIV isolates from a goat and a sheep. No RVFV-derived viral RNA was detected, but 81 seropositive animals including 22 IgM-positive individuals were found. Of these specimens, 61 samples revealed antibodies against RVFV and at least against one of the three orthobunyaviruses. An indirect ELISA based on NRIV/BATV and BUNV derived Gc protein was established as complement to SNT, which showed high performance regarding NRIV, but decreased sensitivity and specificity regarding BATV and BUNV. Moreover, we observed high cross-reactivity among NRIV and BATV serological assays. Taken together, the data indicate the co-circulation of at least BUNV and NRIV in the Mauritanian sheep and goat populations.

## Introduction

Ngari virus (NRIV), Bunyamwera virus (BUNV) and Batai virus (BATV) are members of the Bunyamwera serogroup in the genus *Orthobunyavirus* of the family *Peribunyaviridae*. They are characterized by a tri-segmented (S-, M- and L-segment) enveloped negative-stranded RNA genome. The S-segment encodes the NP and NSs proteins, the M-segment encodes the two glycoproteins Gn and Gc and the NSm protein, and the L-segment codes for the RNA-dependent RNA-polymerase (Elliott, 2014). Sequence analysis showed that NRIV is a natural reassortant resulting from co-infection of BUNV and BATV, as NRIV possesses the M-segment of BATV combined with the S- and L-segments of BUNV (Briese et al., 2006). This reassortment probably led to an increased virulence, which is associated with hemorrhagic fever in humans and ruminants (Dutuze et al., 2018). In contrast, infection with BATV or BUNV is reported to cause only mild flu-like disease in humans (Dutuze et al., 2018). Susceptible vertebrate hosts for BUNV and BATV are ruminants, horses and birds (Hubálek et al., 2014). The BATV strain Chittoor in India caused mild unspecific disease in sheep and goats (Singh and Pavri, 1966), whereas in Europe no disease association in ruminants was described yet, although a high seroprevalence has been determined in Central German goats, sheep and cattle (Ziegler et al., 2018; Cichon et al., 2021). Nevertheless, a German captive harbour seal, which died from encephalitis, tested positive for BATV infection (Jo et al., 2018). In North America, the BUNV strain Cache Valley virus is associated with congenital abnormalities in sheep and other ruminants (Chung et al., 1990; Edwards, 1994). Moreover, in Argentina, BUNV was determined as a causative agent for fatal encephalitis and abortion in horses (Tauro et al., 2015). Likewise, NRIV, BATV and BUNV are transmitted by mosquitoes (Dutuze et al., 2018). For transmission of BUNV, *Aedes aegypti* might be the primary vector (Odhiambo et al., 2014). Additionally, in Argentina, BUNV was isolated from *Ochloretatus scapularis* (Tauro et al., 2015). Infection studies revealed that *Anopheles gambiae Giles* is a competent vector for both, BUNV and NRIV (Odhiambo et al., 2014). Furthermore, NRIV was isolated from various mosquito species in Senegal such as *Aedes argentepunctatus*, *A*. *minutus*, *A*. *vexans*, *A*. *mcintoshi*, *A*. *simpsoni*, *A*. *vittatus*, *A*. *neoafricanus*, *Anopheles coustani*, *A*. *pretoriensis*, *A*. *pharoensis*, *A*. *mascarensis*, *Culex bitaeniorhynchus*, *C*. *tritaeniorhynchus*, *C*. *antennatus*, and *C*. *poicilipes* (Zeller et al., 1996). European mosquito species transmitting BATV are *Anopheles maculipennis s.l*, *A. claviger*, *Coquillettidia richiardii*, *Culex pipiens*, *Ochlerotatus punctor*, *O. communis*, and *Aedes vexans* (Hubálek et al., 2014). Recently, NRIV was detected in ticks [species: *Amblyomma variegatum*, *Rhipicephalus geigyi*, and *Rhipicephalus* (*Boophilus*) spp.] collected from cattle in Guinea (Makenov et al., 2021).

So far, NRIV was isolated only from sub-Saharan Africa, whereas BATV is distributed almost worldwide. Its distribution ranges from Malaysia towards Asian Russia and India and in Europe from Scandinavia towards Italy and Romania (Dutuze et al., 2018). In Africa, the virus was described as Ilesha virus in Sudan, Cameroon, Nigeria, Uganda, and Central African Republic (Hubálek et al., 2014; Dutuze et al., 2018), detected in Mauritania (Gonzalez, 1988) and most recently in Rwanda (Dutuze et al., 2020). BUNV was primarily isolated in several sub-Saharan African countries, such as Uganda, Tanzania, Mozambique, Nigeria, Guinea, South Africa, Democratic Republic of Congo, Senegal, Guinea, Ivory Coast, Nigeria, Cameroon, Central African Republic, Kenya, Uganda, South Africa, Madagascar, and Rwanda (Dutuze et al., 2018, 2020). Moreover, strains of BUNV have been discovered in North America towards Mexico and Argentina (Dutuze et al., 2018). However, the distribution of these viruses might be underestimated since diagnostic capabilities available for orthobunyaviruses in general are limited and the diagnostic approach was primarily based on clinical presentation (Dutuze et al., 2018). Hence, three outbreaks of NRIV were first mistaken for more common aetiologies of hemorrhagic fever, but afterwards were identified as NRIV outbreak. This happened in Sudan in 1988, when an outbreak of hemorrhagic fever was clinically diagnosed as malaria, but subsequently two isolates of NRIV were found and IgM antibodies against NRIV were detected in 7% of patients (Briese et al., 2006). Likewise, NRIV has been isolated twice during concurrent Rift Valley fever (RVF) outbreaks, in Kenya and Somalia in 1997–1998 and in Mauritania in 2010 (Bowen et al., 2001; Eiden et al., 2014). Rift Valley fever virus (RVFV), a member of the *Phenuiviridae* family in the genus *Phlebovirus*, is a zoonotic mosquito-borne virus of major concern throughout Africa (from Egypt towards South Africa) and even emerged in the Arabian Peninsula in 2000 (Linthicum et al., 2016). Clinical symptoms of an infection with RVFV closely resemble the symptoms caused by NRIV infection: in mild cases human patients show flu-like febrile illness, whereas in 1–2% the infection can develop into severe hemorrhagic fever, encephalitis, and retinitis (Chevalier, 2013). RVFV and NRIV not only present many similarities in their clinical manifestations, but as well share the same ecological distribution, and co-circulate within the same vector and vertebrate host ranges (Dutuze et al., 2018). Outbreaks of RVF characteristically coincide with episodes of unusually heavy rains and extensive flooding in areas normally arid, which were also observed in Sudan in 1988 and Somalia/Kenya in 1997–1998 during the NRIV outbreaks (Briese et al., 2006). Heavy rainfall and consecutive flooding induce mass hatching of *Aedes* species from infected eggs and subsequently of *Culex* species, which then infect humans and ruminants (Linthicum et al., 2016).

In 2015/16, Mauritania experienced the most recent outbreak of RVF, affecting sheep and goats (OIE) as well as humans (Boushab et al., 2016). To investigate the prevalence of RVFV and possible co-infection with NRIV, BATV, and BUNV in the small ruminant population, 492 serum samples of goats and sheep were collected in 2015 in southwestern Mauritania and analyzed by quantitative real-time RT-PCR (qRT-PCR), serum neutralization tests (SNT) specific for each virus, and commercial enzyme-linked immunosorbent assays (ELISA). Additionally, we implemented ELISAs based on the glycoprotein Gc of each NRIV, BATV, and BUNV.

## Materials and Methods

### Sample Collection and Workflow

**Figure 1:**
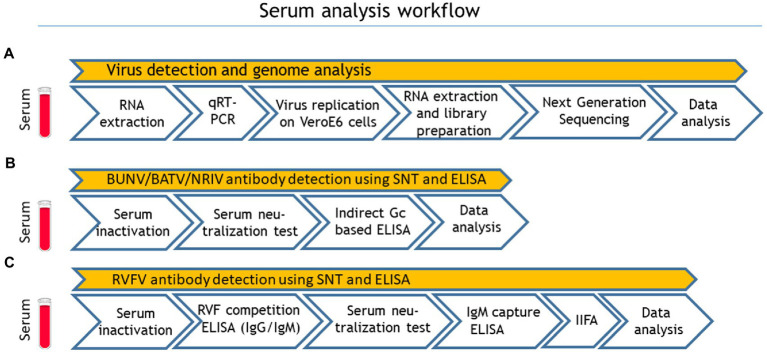
Sample processing workflow: **(A)** virus detection and genome analysis, **(B)** Bunyamwera virus (BUNV), Batai virus (BATV) and Ngari virus (NRIV) related antibody detection and **(C)** Rift valley fever virus (RVFV) related antibody detection. Quantitative real-time RT-PCR (qRT-PCR), serum neutralization test (SNT) and indirect immunofluorescence assay.

A total number of 492 apparently healthy sheep and goats were sampled in eight different governorates of Mauritania (Inchiri, Hodh Ech Chargui, Tagant, Assaba, Trarza, Guidimakha, Brakna, Hodh El Gharbi) in 2015 (Table 1). Serum samples were kept frozen at −20°C for further investigations. After transportation to Germany, all samples were first analyzed for viral RNA with qRT-PCR. For serological testing, the sera were inactivated with 1:1 phosphate-buffered saline (PBS) Tween at 56°C for 30 min. For detection of antibodies against NRIV, BATV, and BUNV, samples were subjected to SNTs and additionally to indirect Glycoprotein Gc-based ELISA. The serological investigation for RVFV-specific antibodies was performed with a competition ELISA (IDvet, Grabels, France) followed by SNT (Rissmann et al., 2017b). Samples with positive and inconclusive results in the ELISAs, were further tested with the RVF IDvet IgM capture ELISA (IDvet, Grabels, France). In case of divergent results in the ELISA and SNT, a final assessment was performed with an adapted commercial immunofluorescence assay (IIFA; Euroimmun, Lübeck, Germany). The complete workflow is seen in Figure 1.

**Table 1:** Samples ordered by species and region.

Region	Species	No of samples	Age	Date of sample collection
Inchiri	goat	50	1–5	February 2015
	sheep	29	1–4	November 2015
Chargui	goat	41	1–10	July 2015
	sheep	40	1–5	July 2015
Gharbi	sheep	81	1–7	August 2015
	not available (NA)	4	not available (NA)	October 2015
Tagant	goat	54	1–3	September + October 2015
	sheep	5	not available (NA)	October 2015
Assaba	goat	28	1–6	September 2015
	sheep	32	1–5	October 2015
Trarza	goat	12	1–4	October 2015
	sheep	19	1–3	October 2015
Guidimakha	goat	40	1–4	September 2015
	sheep	34	1–4	September 2015
Brakna	goat	9	3–6	September 2015
	sheep	13	3–5	September 2015
	not available (NA)	1	not available (NA)	September 2015

### Quantitative Real-Time RT-PCR

RNA isolation was performed using the Nucleo^®^Mag VET Kit (Macherey-Nagel, Düren, Germany) according to the manufacturer’s instructions. The samples were processed in a pool of five samples. As internal extraction control, MS2 bacteriophage was added to each serum pool before the extraction process. Control RNA detection was performed with primers MS2F (5'-CTC TGA GAG CGG CTC TAT TGG T-3') and MS2R (5'-GTT CCC TAC AAC GAG CCT AAA TTC-3') and MS2P (HEX-TCAGACACGCGGTCCGCTATAACGA-BHQ1; Ninove et al., 2011). The qRT-PCR for RVFV was carried out according to an adapted multiplex qRT-PCR protocol (Wernike et al., 2015) using the AgPath-ID^™^ One-Step RT-PCR Kit (Applied Biosystems, Foster City, United States). The protocol allows the simultaneous detection of RVFV, foot and mouth disease virus (FMDV) and NRIV/BUNV, and uses the following primers: RVF-forw (5'-TGA AAA TTC CTG AGA CAC ATG G-3'), RVF-rev (5'-CTT CCT TGC ATC TGA TG-3') and RVFV probe (FAM-CAATGTAAGGGGCCTGTGTGGACTTGTG-BHQ1) for RVFV, FMD-IRES-4.1F (5'-TAA CAW GGA CCC RCS GGG CC-3'), FMD-IRES-4R (5'-TGA AGG GCA TCC TTA GCC TG-3') and FMD-IRES probe (Texas Red-CAT GTG TGC AAY CCC AGC ACR G-BHQ2) for FMDV. For detection of NRIV/BUNV S-segment, novel primers/probes were used: Bunyam_F (5'-GCT GGA AGA TTA CTG TAT ATA C-3'), Bunyam_R (5'-CAA GGA ATC CAC TGA GGC GGT G-3') and Bunyam_P (HEX-AAC AAC CCA GTT CCT GAC GAT GGT C-BHQ2). The final concentrations in the used AgPath-ID^™^ One-Step RT-PCR Kit (Applied Biosystems, Foster City, United States) were 0.4 μm for primer and 0.08 μm for the probe, respectively. For each reaction, 2.5 μl of RNA, 5 pmol of both forward and reverse primer and 0.625 pmol of the probe were used in a total volume of 12.5 μl. PCR reaction condition was used as follows: 48°C for 10 min, 95°C for 10 min and 44 cycles at 95°C for 15 s, and 60°C for 45 s. Finally, a qRT-PCR assay in a total volume of 12.5 μl targeting the BATV S-segment (Jöst et al., 2011) was performed.

### Phylogenetic Analysis

Phylogenetic analysis of full-length sequences was done with Geneious Tree Builder using Neighbor-Joining analysis and genetic distances were calculated using the Tamura-Nei Method. Bootstrap values >80 are displayed at nodes. A sequence of a La Crosse virus, Human/78 strain (accession number: AF528165-167) was used as outgroup to root the tree. Sequence alignments were performed with Clystal W.

### Sequencing

Vero E6 cells (CRL-1586, Collection of Cell Lines in Veterinary Medicine, Friedrich-Loeffler-Institut, Germany) were inoculated with 100 μl PCR positive serum samples and assayed for virus replication and cytopathic effects (CPE). After appearance of CPE after 3–4 days, viral RNA was extracted from the supernatant and subjected to a next-generation sequencing workflow (Wylezich et al., 2018). All assembly and mapping analyses were conducted with the 454/Roche Genome Sequencer FLX software suite v3.0 (Roche, Mannheim, Germany) leading to three final datasets (lib02232-lib02234). As part of the assemblies and mappings, adapter and quality trimming were performed using the available adapter sequences’ default software settings. First, a partial dataset (25.000 reads) of lib02232 was assembled and the genome segments identified in the obtained contigs. For quality control, the sequences were visually inspected for base qualities and the ORFs were detected using emboss getorf (Rice et al., 2000). Subsequently, the sequences were analyzed using blast (Altschul et al., 1990) with the suitable NCBI nt (for nucleotide sequence analyses) and nr (for amino acid sequence analyses) databases. Afterwards, the quality checked sequences of lib02232 were used to map the full datasets along these to obtain the three segment sequences of lib02232 (total number of reads 2.228.328, after trimming 2.230.666, matching reads 519.568, resulting average depth approx. 24.900), lib02233 (total 617.530, trimmed 617.664, mapped 270.410, avg. depth 6,500) and lib02234 (total 570.042, trimmed 570.434, mapped 325.364, avg. depth 7,700), which were again quality checked as described. The obtained full-length recovered genome sequences of BUNV (accession no. MT731755 - MT731757) and NRIV (accession Nr. MT747972 - MT747974 and MT747975 - MT747977) were submitted to GenBank.

### Serum Neutralization Test

The SNT for detecting BATV neutralizing antibodies was performed using BATV strain 53.2 (acc. no. HQ455790, kindly provided by J. Schmidt-Chanasit, BNITM Hamburg, Germany) as already described by Seidowski et al. (2010) and Ziegler et al. (2018). Modifications were made using Vero E6 cells (CRL-1586) and applying an incubation time of 6 days. Similarly, the SNTs for detecting NRIV and BUNV neutralizing antibodies were performed using a Mauritanian NRIV strain (accession nos. KJ716848–716850) and BUNV ATCC^®^ VR87^™^ (kindly provided by J. Schmidt-Chanasit, BNITM Hamburg, Germany). Briefly, a virus concentration of 100 TCID_50_/well was added to each sample running in duplicate at a starting serum dilution of 1:10. Cytopathic effects were seen 4–6 days post infection. The SNT for detecting RVFV neutralizing antibodies was performed as described in the OIE Terrestrial Manual 2014 (OIE, 2020). Briefly, 100 TCID_50_ of RVFV (MP-12 vaccine strain) were added to serial two-fold diluted sera and 3×10^5^ Vero 76 cells/ml (Collection of Cell Lines in Veterinary Medicine, Friedrich-Loeffler-Institut, Germany) were added to each well. Plates were incubated at 37°C, 5% CO_2_ for 6 days. The neutralizing antibody titer of the samples was defined as the 50% neutralization dose (ND_50_). Positive results for neutralizing antibodies were confirmed when the ND_50_ was 10 or higher. Positive serum controls were obtained from a sheep immunised with inactivated NRIV virus, a goat immunised with inactivated BUNV virus and a rabbit immunised with inactivated BATV. Negative control serum was derived from an untreated goat.

### Recombinant Glycoprotein Gc

Synthetic genes encompassing the domains GI and GII of the glycoprotein Gc were produced by Eurofins (Munich, Germany) based on a partial BATV sequence (acc. no. HQ455791, nucleotide position 601–1,650), the corresponding NRIV sequence (acc. no. KJ716849, position 1,552–2,505) and a BUNV sequence (acc. no. M11852, position 1,479–2,534). The corresponding protein sequences were aligned using CLUSTALW within the geneious^®^ software platform (Auckland, New Zealand). The alignment is depicted in Supplementary Figure S1. All three sequences were optimized for expression in *E. coli*. The sequence codes for putative domains I and II of glycoprotein Gc and were cloned into *E. coli* expression vector pET21 a using 5’ *BamH*I and 3’ *Xho*I restrictions sides and expressed in BL21-Lys cells. Expression of the recombinant proteins and purification by nickel chelating agarose were carried out under denaturing conditions as described previously (Jäckel et al., 2013a). Finally, the proteins were dialyzed against 0.05 M carbonate-bicarbonate buffer pH 9.6 and checked in SDS-PAGE and Coomassie-staining.

### Indirect (NRIV/BATV/BUNV) ELISA

For serological testing by ELISA, the collected serum samples were inactivated with 1:1 PBS Tween at 56°C for 30 min. The three indirect in-house ELISAs were based on a partial recombinant glycoprotein Gc of either NRIV or BATV or BUNV which were used for coating immunoplates in a dilution of 2 μg/ml in 0.05 M carbonate–bicarbonate buffer pH 9.6 (100 μl diluted antigen per well). Protocol parameters, dilutions, optimal reagent concentrations and the selection of immunoplates were determined by standard checkerboard titration and choosing the combination with the highest difference in the optical density (OD value) between positive and negative controls. After incubation of the coated immunoplates at 4°C overnight, plates were washed three times with 300 μl washing buffer, containing PBS pH 7.2 and 0.1% Tween 20. After blocking with 200 μl/well 10% skim milk powder (DIFCO^™^) diluted in PBS for 1 h at 37°C in a moist chamber, ruminant field sera diluted 1:20 for NRIV ELISA and 1:10 for BATV and BUNV ELISA in PBS containing 2% skim milk were added in duplicate to the plates. As positive control, polyclonal hyperimmune sheep serum were diluted 1:20 for NRIV ELISA and 1:10 for BATV and BUNV ELISA, respectively. A volume of 100 μl of each sample and control was added to the plates. After incubation at 37°C for 1 h in a moist chamber, plates were again washed three times with washing buffer. A volume of 100 μl per well of horseradish peroxidase conjugated Protein G (Calbiochem, San Diego, CA) diluted 1:5000 in dilution buffer was then added to the plates and incubated again for 1 h, as described before. After a final washing step, 100 μl per well of 2,20-azinodiethylbenzothiazoline sulfonic acid substrate (ABTS, Roche, Mannheim, Germany) was added and plates were incubated for 30 min at room temperature in the dark. The reaction was stopped by adding 1% sodium dodecyl sulphate and OD value was determined at 405 nm. In case of BUNV ELISA, blocking milk and dilution buffer of IDvet were used following the same protocol as described above. The results were expressed as percentage of the positive control serum (PP value) using the following formula: (mean OD of duplicate test serum/median OD of duplicate positive control) *100. Cut-off value, sensitivity and specificity of the indirect ELISAs were determined in correlation to the corresponding SNT results using the receiver operating characteristic analysis (ROC analysis) with regard to the criterion “Maximization of sensitivity and specificity.” Finally, to determine the accordance between the SNTs and the ELISAs among each other, the kappa coefficient was calculated.

### IDvet RVF Competition ELISA and IDvet RVF IgM Capture ELISA

All samples were analyzed in the commercial ID Screen^®^ RVFV competition multispecies ELISA (IDvet, Grabels, France) according to the manufacturer’s instructions. Both IgG and IgM antibodies are detected indistinguishably. Samples that gave positive or inconclusive results in the competition ELISA, were further tested with the ID Screen^®^ Rift Valley Fever IgM capture ELISA (IDvet, Grabels, France) according to the manufacturer’s description.

### Indirect Immunofluorescence

Samples that gave divergent results in ELISAs and SNT were further analyzed with a RVFV in-house IIFA according to a previously published protocol (Jäckel et al., 2013b) using the commercial RVFV immunofluorescence slides from Euroimmun (Lübeck, Germany). The detection of antibodies was realized with species-specific Cy3 labelled secondary antibodies (donkey anti-sheep, donkey anti-goat) from dianova (Hamburg, Germany), in a 1:200 dilution.

### Statistical Analysis

The estimated prevalence and 95% confidence intervals (95% CI) were calculated using the calculation tool of Epitools.^1^ Calculations for ROC-analysis were performed using the software (R Core Team, 2020) and the R package OptimalCutpoints (Lopez-Raton et al., 2014).

## Results

Among the 492 serum samples tested consecutively for NRIV, BUNV, BATV and RVFV by qRT-PCR assays, 10 samples were tested positive for BUNV/NRIV-derived RNA. Subsequent sequencing revealed two NRIV isolates and two BUNV isolates (Table 2). The positive serum samples originated from one goat and three sheep from Trarza, Guidimakha and Brakna governorates. No RVFV or BATV genome was detected (Table 2).

**Table 2:** Summary of results from multiplex qRT-PCR (detecting NRIV, BATV, and BUNV) and sequencing.

Sample ID	Species	Region	CT Multiplex	CT Singleplex	Sequencing
			(BUNV S)	(BUNV S)	
MR 393/15 SR	goat	Trarza	no CT	34.69	NRIV
MR 410/15 SR	sheep	Guidimakha	32.33	32.04	
MR 411/15 SR	sheep	Guidimakha	26.96	26.72	NRIV
MR 471/15 SR	sheep	Brakna	28.86	28.82	
MR 478/15 SR	sheep	Brakna	27.19	26.85	BUNV
MR 479/15 SR	sheep	Brakna	30.51	30.21	
MR 487/15 SR	sheep	Brakna	27.4	25.05	BUNV
MR 492/15 SR	goat	Brakna	no CT	34.47	
MR 495/15 SR	sheep	Brakna	30.48	28.16	
MR 496/15 SR	sheep	Brakna	33.98	31.21	

**Figure 2:**
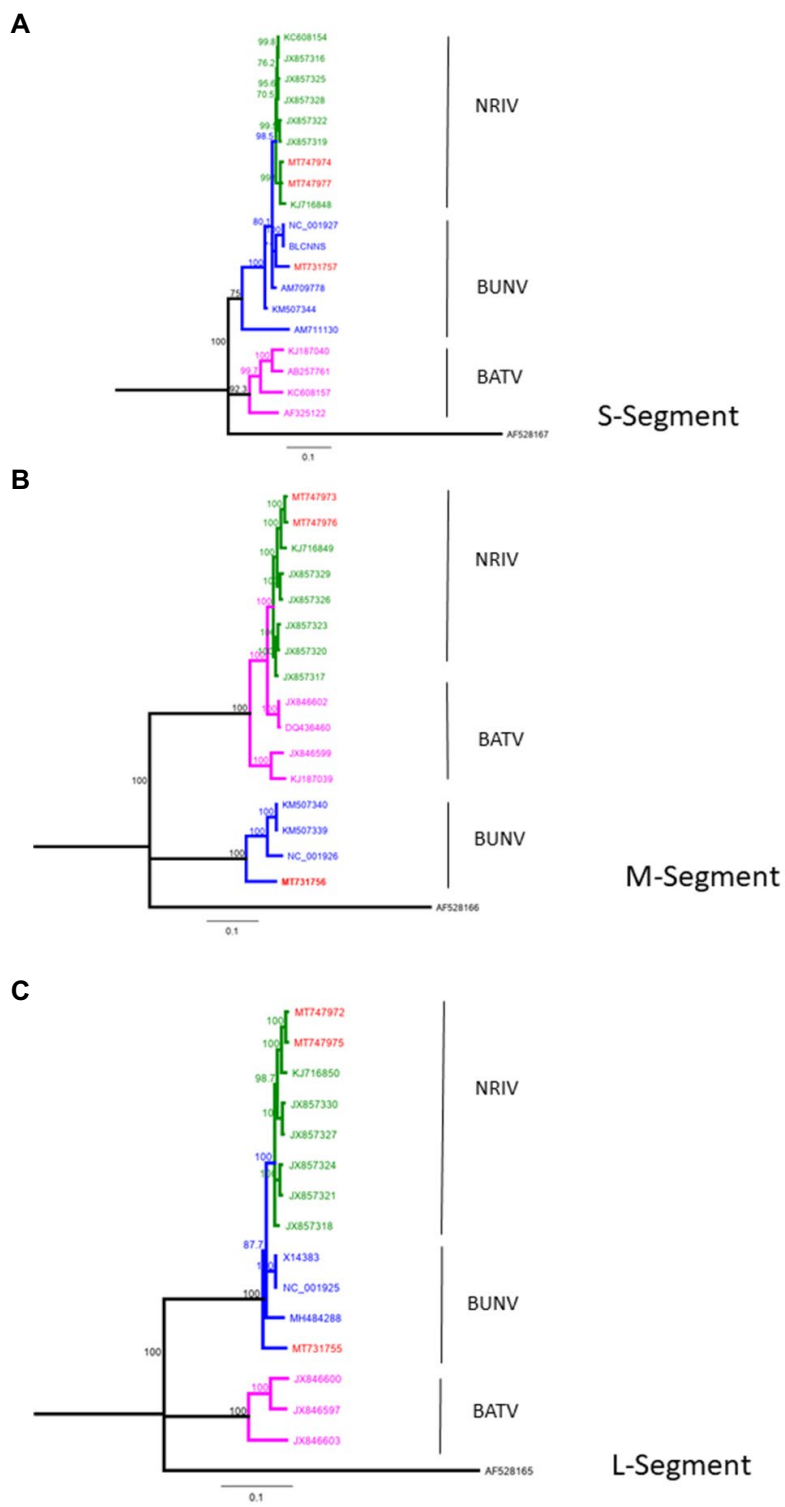
Phylogeny of complete BUNV, BATV and NRIV sequences for **(A)** small (S)-Segment, **(B)** medium (M)-Segment and **(C)** large (L)-Segment compared with sequences obtained from goat and sheep in Mauritania from 2015 (red lines). Green lines indicate NRIV related, blue lines BUNV related and purple lines BATV related sequences. The tree was constructed by Neighbor-Joining analysis and genetic distances were calculated using the Tamura-Nei Method. The scale bar indicates the number of nucleotide substitutions per site. Numbers before the nodes denote bootstrap values ≥80%. The tree was rooted to the sequence of La Crosse virus, Human/78 strain (accession number: AF528165-167).

From four animals, full-length genome sequences of the S-, M-, and L-segments were recovered. Phylogenetic analysis of complete genomes revealed that two isolates clustered to NRIV and two isolates to the BUNV group (Figure 2). NRIV was isolated from one goat of the Trarza region and from a sheep of the Guidimakha region. Both isolates show high similarity among each other with 4 (99,6% sequence identity), 32 (99,8% sequence identity) and 31 (99,5% sequence identity) nucleotide differences corresponding to S-, M-, and L-segment. In addition, they exhibited high similarity to a NRIV isolate recovered from a goat of the Adrar region in 2010. BUNV was isolated from two sheep of the Brakna region. The sequences were identical, indicating the co-infection of two individuals from one flock with the same isolate. The strain showed highest similarity to isolates from Kenya (43 nucleotide differences), from Dakar (161 nucleotide differences) and the BUNV prototype (279 nucleotide differences) regarding S-, M- and L-segments.

**Figure 3:**
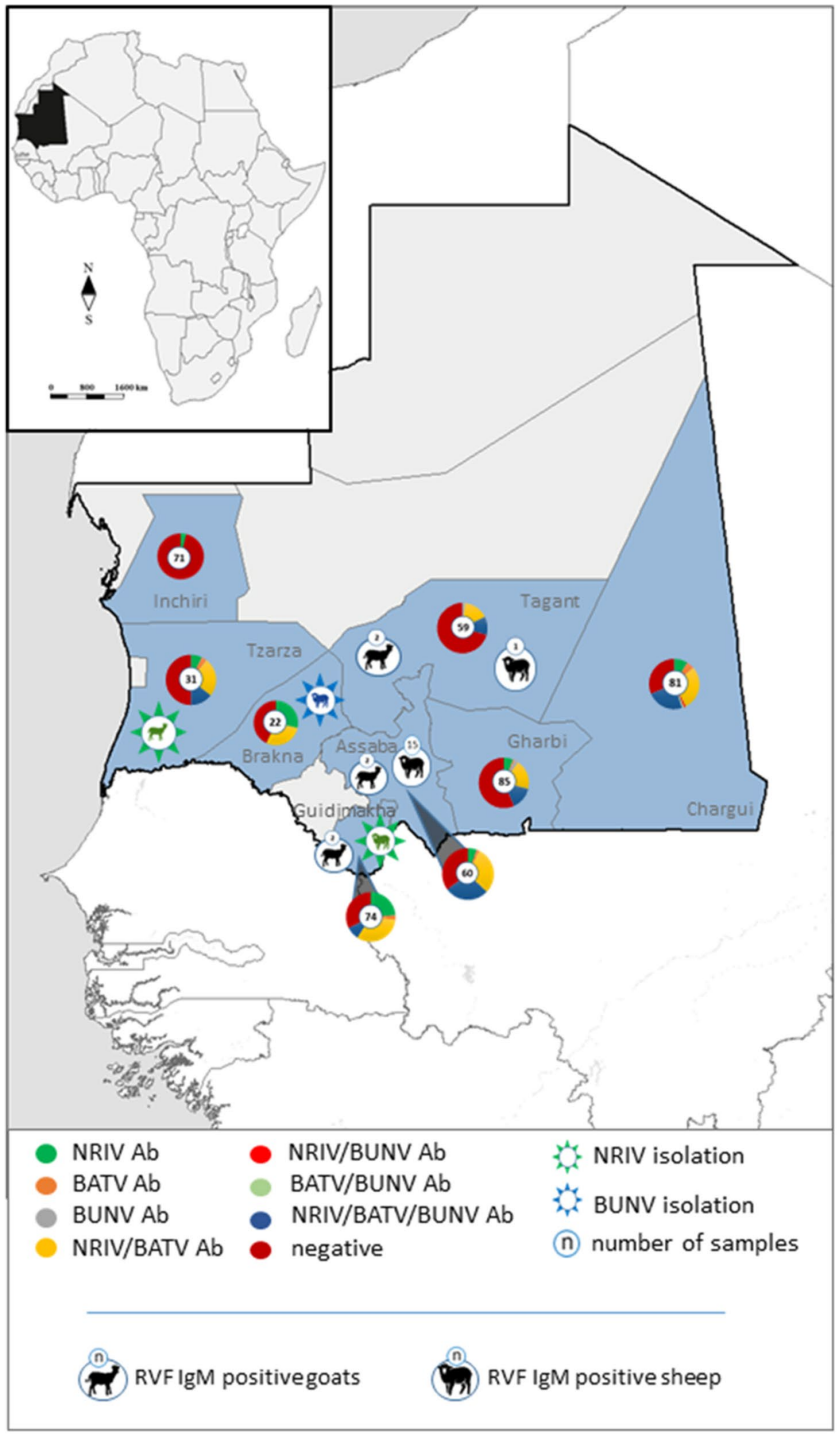
Geographical distribution of specific and non-differentiable antibodies against NRIV, BATV and BUNV in the investigated Mauritanian small ruminants depicted in pie charts. Isolated NRIV (green coloured) and BUNV (blue coloured) are highlighted in star embedded animals. RVFV IgM positive sheep and goats are displayed in black symbols.

**Figure 4:**
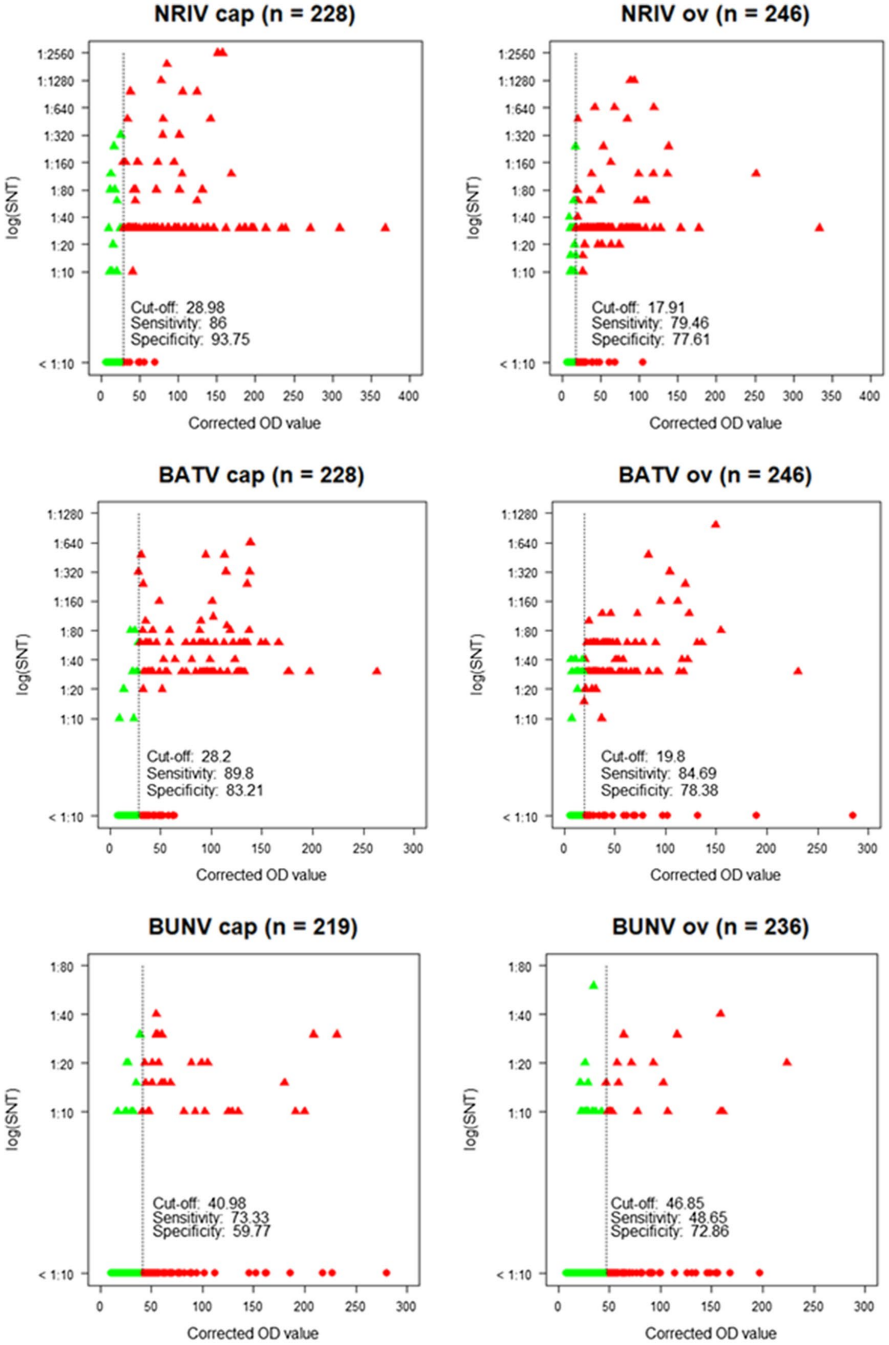
Corrected OD values of the ELISA in relation to neutralization titers [log (SNT)] of the SNT showing cut-off, sensitivity and specificity for each species (cap = goat, ov = sheep) and each virus. Green dots: SNT and ELISA negative samples. Green triangles: SNT positive, but ELISA negative samples. Red triangles: SNT and ELISA positive samples. Red dots: SNT negative, but ELISA positive samples.

The detection of antibodies directed against all three viruses was determined by screening the samples consecutively by NRIV-, BATV- and BUNV-derived SNTs. Due to low sample volume, only 458 out of 492 sera could be comprehensively analyzed in SNTs and indirect ELISAs Sera showing a ND_50_ higher than 1:30 were retested to determine the endpoint values. Different neutralizing antibodies detected simultaneously in one sample were considered specific if individual ND_50_ was four times higher compared to the other(s). Within the meaning of that definition, in 41 out of 458 samples NRIV-specific antibodies were detected, leading to a total prevalence of 8.95% (Table 3). BATV-specific antibodies were found in nine samples (prevalence of 1.97%), and BUNV-specific antibodies in only three samples (prevalence of 0.66%). In 97 sera, similar antibody titers against NRIV and BATV were found (prevalence of 21.18%), whereas only one sample was positive for NRIV as well as BUNV antibodies and another one for BATV as well as BUNV antibodies corresponding to a prevalence of 0.22%. Finally, 62 sera were tested positive in all three SNTs leading to a prevalence of 13.54%. Interestingly, the NRIV-positive samples showed the highest values with antibody titers up to 1:2560. In BATV-based SNT, only one sample reached a titer of up to 1:960. Regarding BUNV-specific SNT, the titer did not exceed 1:40, but mostly ranged between 1:10 and 1:20. The geographical distribution of seropositive samples is displayed in Figure 3. To further analyze the antibodies, they were tested in an indirect ELISA for reactivity against glycoprotein Gc of NRIV, BATV or BUNV, which are the main targets for virus neutralisation. The resulting OD values were compared with the results of the homologous SNT, which is used as reference and gold standard. Goat and sheep samples were evaluated separately by ROC analysis to determine specificity and sensitivity (Figure 4). All individual values are compiled in the Supplementary Table.

**Table 3:** Serological analysis of the Mauritanian serum samples using SNT.

(A)			NRIV SNT positive				
Species/Region	No of samples	Goat	Sheep	Not available (NA)	Total	Prevalence (%)	95% CI
Inchiri	77	2	1	0	3	3.9	1.3–10.8
Chargui	80	1	5	1	7	8.8	4.3–17.0
Gharbi	83	0	5	0	5	6.0	2.6–13.3
Tagant	51	0	0	0	0	0.0	0.0–7.0
Assaba	54	3	0	0	3	5.6	1.9–15.1
Trarza	28	1	1	0	2	7.1	2–22.7
Guidimaka	71	11	6	0	17	23.9	15.5–35.0
Brakna	14	2	2	0	4	28.6	11.7–54.7
total	458	20	20	1	41	9.0	6.7–11.9
**(B)**			**BATV SNT positive**				
Inchiri	77	0	0	0	0	0.0	0.0–4.8
Chargui	80	2	2	0	4	5.0	2.0–12.1
Gharbi	83	0	0	1	1	1.2	0.2–6.5
Tagant	51	0	0	0	0	0.0	0.0–7.0
Assaba	54	1	0	0	1	1.9	0.3–9.8
Trarza	28	0	1	0	1	3.6	0.6–17.7
Guidimaka	71	2	0	0	2	2.8	0.8–9.7
Brakna	14	0	0	0	0	0.0	0.0–21.5
total	458	5	3	1	9	2.0	1.0–3.7
**(C)**			**BUNV SNT positive**				
Inchiri	77	0	0	0	0	0.0	0.0–4.8
Chargui	80	0	0	0	0	0.0	0.0–4.6
Gharbi	83	0	2	0	2	2.4	0.7–8.3
Tagant	51	1	0	0	1	2.0	0.4–10.3
Assaba	54	0	0	0	0	0.0	0.0–6.6
Trarza	28	0	0	0	0	0.0	0.0–12.1
Guidimaka	71	0	0	0	0	0.0	0.0–5.1
Brakna	14	0	0	0	0	0.0	0.0–21.5
total	458	1	2	0	3	0.7	0.2–1.9
**(D)**			**RVFV SNT positive**				
Inchiri	77	1	3	0	4	5.2	2.0–12.6
Chargui	80	1	0	0	1	1.3	0.2–6.8
Gharbi	83	0	11	2	13	15.7	9.4–25.0
Tagant	51	14	1	0	15	29.4	18.7–43.0
Assaba	54	9	20	0	29	53.7	40.6–66.3
Trarza	28	4	0	0	4	14.3	5.1–31.5
Guidimaka	71	6	3	0	9	12.7	6.8–22.4
Brakna	14	3	3	0	6	42.9	21.4–67.4
total	458	38	41	2	81	17.7	14.5–21.4

***(A)*** *NRIV*, **(B)** *BATV*, **(C)** *BUNV*, *and* **(D)** *RVFV specific antibodies*.

The NRIV Gc-based ELISA displayed a specificity of 93.75 and 77.61% for goats and sheep, respectively, and a corresponding sensitivity of 86 and 79.46%. BATV Gc-based ELISA exhibited a specificity of 83.21% (goat) and 78.38% (sheep), and a sensitivity of 84.69 and 89.8%, respectively. In the case of the BUNV Gc-based ELISA, significantly lower values for specificity (72.86 and 59.77%) and sensitivity (48.65 and 73.33%) were found. Using the calculated cut-offs, the NRIV Gc-based ELISA detected 13 positive samples, the BATV Gc-based ELISA 24 seropositive specimens and the BUNV-based ELISA 37 positive samples. 92 specimens showed reactivity in all three ELISAs, whereas 87 samples were positive in the NRIV/BATV-based ELISAs. 15 samples showed reactivity against NRIV and BUNV Gc and 14 samples reactivity against BUNV/BATV Gc.

The degree of accordance between the serological assays of the three viruses was calculated by the kappa coefficient. Between SNT specific for NRIV and SNT specific for BATV for both goat- and sheep-derived samples, the coefficient revealed an almost perfect agreement (*κ* = 0.90, value of *p* < 2.2e-16, 95% CI 0.84–0.96, and *κ* = 0.86, value of *p* < 2.2e-16, 95% CI 0.80–0.93). Between NRIV Gc-based ELISA and BATV Gc-based ELISA (for goat and sheep) the kappa coefficient showed a substantial agreement (*κ* = 0.73, value of *p* < 2.2e-16, 95% CI 0.64–0.82, and *κ* = 0.71, value of *p* < 2.2e-16, 95% CI 0.62–0.80). In contrast, the comparison of BUNV Gc ELISA or specific SNT only showed fair to moderate agreement with BATV and NRIV serological tests (data not shown).

Finally, all samples were analyzed for antibodies against RVFV with a commercial species independent ELISA which revealed 84 antibody positive samples. All positive sera were verified by the SNT, which confirmed seropositivity in 81 cases (Table 3). Hence, 81 out of 458 sera were determined antibody positive for RVFV corresponding to a prevalence of 17.69% (Table 3). Highest antibody titer were found in the regions Tagant, Assaba, and Brakna (29.41, 53.7, and 42.86%, respectively). Since the competition ELISA does not distinguish between IgG and IgM antibodies, all seropositive samples were tested by the IDvet IgM capture ELISA. Hereby, 22 samples revealed RVFV IgM antibodies resulting in a prevalence of 4.80%, of which 16 samples were collected in Assaba alone. Interestingly, of these samples only 4 were exclusively positive for RVF IgM, but the remaining IgM positive were found in BATV/NRIV positive individuals. The distribution of RVFV IgM positive and NRIV and BUNV positive samples is shown in Figure 2.

## Discussion

A co-infection of the ruminant population with RVFV and NRIV was already described in Kenya and Somalia in 1997–1998 (Bowen et al., 2001) and in Mauritania in 2010 (Eiden et al., 2014). In Kenya, 23% of the hemorrhagic fever cases were diagnozed as infected with RVFV and 27% as infected with NRIV (Bowen et al., 2001). In Mauritania, the RVF outbreak caused 63 human infections (El Mamy et al., 2011) and 57 infected small ruminants out of 93 tested animals (Jäckel et al., 2013b). Moreover, Dutuze et al. observed the co-circulation of RVFV with BUNV and BATV in the ruminant population in Rwanda (Dutuze et al., 2020). In Mauritania, the most recent RVF outbreak started in September 2020 in Assaba, Tagant, Brakna, Trarza, Hodh El Gharbi and Hodh Ech Chargui and affected camels, small ruminants and cattle. Moreover, 25 human deaths have been reported (World Health Organization, 2020). Five years earlier, 31 human patients were infected in the same region, some of whom were suffering from hemorrhagic and neurologic manifestations (Boushab et al., 2016). Furthermore, in three regions in southern Mauritania (Brakna, Tagant, and Assaba) a total of 19 infected sheep and goats were detected and stamped out as part of outbreak control (OIE). In these three and in additional five regions (Brakna, Tagant, Assaba, Tasiast in Inchiri region) Hodh Ech Chargui, Trarza, Guidimakha, and Hodh El Gharbi, our project partners collected 492 samples of sheep and goats before and at the beginning of the outbreak in the year of 2015. The serum samples were investigated comprehensively for RVFV and the orthobunyaviruses NRIV, BATV, and BUNV to determine if these viruses were co-circulating in Mauritania again.

Viral RVFV RNA was not detected, but 17.69% of the investigated animals showed IgG/IgM antibodies. In comparison, in 2012/13 during an inter-epidemic phase, Rissmann et al. reported a much lower prevalence of 3.8% in 497 investigated small ruminants in Mauritania (Rissmann et al., 2017a). Thus, we observed a clear increase in the overall RVFV antibody prevalence in the small ruminant population in the year of 2015. If the regions are viewed separately, the tested animals from Brakna, Tagant, and Assaba developed the highest antibody titers (29.41, 53.70, and 42.86%, respectively). These were the exact three regions affected by the RVF outbreak in OIE (2020). The OIE reported the first RVFV positive case in Assaba in mid October (OIE). At the same time, we collected serum samples of 32 sheep, of which 16 samples showed RVFV-specific IgM antibodies, underlining the circulation of the virus in Assaba. The goat samples from Assaba were taken 1 month earlier and tested positive for IgM antibodies in only one case. Likewise, in the other investigated regions, only few IgM-positive animals were detected. Therefore, the overall prevalence of IgM antibodies was just 4.80%. As already mentioned, viral RVFV RNA was not detected, but the molecular analysis indicated the presence of NRIV RNA in 10 samples collected in Brakna, Trarza, and Guidimakha. The subsequent sequencing revealed two positive samples for NRIV and two positive samples for BUNV from one goat and three sheep.

Both NRIV- and both BUNV-positive samples were negative for RVFV RNA as well as for RVFV-specific antibodies. However, a total of 61 NRIV, BATV and/or BUNV seropositive samples also revealed antibodies against RVFV, indicating a co-circulation of these viruses in the animal population in Mauritania. Since the RVFV sequence is highly divergent from the deduced orthobunyavirus sequences, cross-reactivity is highly unlikely but needs to be substantiated.

The serological investigation for orthobunyaviruses revealed NRIV-specific antibodies in 41 out of 458 tested samples corresponding to a prevalence of 8.95%, BATV-specific antibodies in nine samples leading to a prevalence of 1.97%, and BUNV-specific antibodies in only three sera (prevalence of 0.66%). However, the results need to be interpreted carefully, since for a large part of seropositive samples an unambiguous determination was not possible. In a total of 161 samples, the SNT of at least two viruses detected neutralizing antibodies. In most cases, the SNT based on NRIV and the SNT based on BATV showed undistinguishable high antibody titers (97 samples corresponding to a prevalence of 21.18%). The percentage of NRIV neutralizing antibodies against BATV and NRIV in each individual sample is shown in Supplementary Figure S2 and indicates the high number of double-positive samples. The high cross-reactivity between NRIV and BATV induced antibodies is based on high sequence identity of NRIV and BATV derived Gc proteins. Alignment of three Gc protein (Domains I and II, Supplementary Figure S1) showed 89.5% sequence identity between BATV and NRIV, but only 45,1% and 46,1% with BUNV glycoprotein. For this reason, the specificity was selected *via* a cut-off with a fourfold higher activity compared to the other two viruses in order to be able to reliably determine a virus-specific reactivity. Factor 4 was taken from previous flavivirus studies in which flaviviruses (particularly from the same serocomplex) can be identified by determining the virus with the highest neutralizing capacity and at least a fourfold difference in titer (Yeh et al., 2012). A total of 62 samples were positive in all three SNTs (prevalence of 13.54%). Thereby, the antibody titer in the BUNV-specific SNT did not exceed a titer of 1:40, but mostly ranged between 1:10 and 1:20. In contrast, in BATV- and NRIV-based SNTs, the antibody titer reached up to 1:1280 or even 1:2560. Apparently, BUNV is less immunogenic and induces a less neutralizing activity. At least, BUNV cross-reacts less with NRIV and BATV than the latter among themselves. A limited cross-reaction between BUNV and BATV was already described by Hunt and Calisher, who investigated the antigenic relationship of 23 strains of Bunyamwera serogroup viruses by plaque reduction neutralization test (Hunt and Calisher, 1979). A further indication for a low neutralizing activity of BUNV is that twice as many samples were positive in the BUNV Gc-based ELISA than in the BUNV SNT (158 ELISA-positive and 67 SNT-positive samples). This also explains the low sensitivity and specificity of the ELISA calculated in correlation to the SNT (48.65% for sheep and 73.33% for goats, and 72.86% for sheep and 59.77% for goats, respectively). The low prevalence of BUNV-seropositive animals determined by the SNT is surprising since we proved the presence of BUNV in the animal population by isolating the virus from two sheep, and therefore expected to detect a stronger immune response in the investigated animals. Similar to the SNT, the ELISAs do not allow an unambiguous differentiation between NRIV, BATV, and BUNV for each sample. Of 458 samples, 87 samples were equally positive in the NRIV Gc-based ELISA and the ELISA based on BATV Gc, whereas a total of 37 samples exhibited antibodies against only one of the viruses. The strong agreement of NRIV- and BATV-based SNTs and ELISAs is confirmed by the kappa coefficient, which for the SNT is *κ* = 0.90 (for goats) and *κ* = 0.86 (for sheep), and for the ELISA *κ* = 0.73 (for goats) and *κ* = 0.71 (for sheep). The high cross-reactivity among the serological tests impedes the determination of whether only one of the viruses has induced the antibody response or a co-infection is present. Neutralizing antibodies are induced by the surface glycoproteins which are encoded by M-segment of the virus (Briese et al., 2006). The M-segment of NRIV closely resembles that of BATV, showing only 11 and 5% differences in nucleotide or deduced amino acid sequence, respectively. This limited divergence is mainly observed in the N-terminal portion of Gc around the conserved potential trypsin cleavage site (Briese et al., 2006). Thus, the specific differentiation between NRIV and BATV antibodies by Gc ELISA or SNT is not possible, but the strong accordance among the assays offers the advantage to use the BATV SNT instead of the NRIV SNT and hereby enables to work in a lower biosafety containment facility.

Overall, we could demonstrate that NRIV and BUNV were co-circulating in Mauritania during the RVF outbreak in 2015/16. Prevalence studies for NRIV, BATV and BUNV are complicated by high cross-reactivity, especially between NRIV and BATV serological assays. Future attempts might consider establishing tests that are based on the nucleoprotein NP to distinguish BATV and NRIV induced antibodies to finally attain a clear serological differentiation of infections by NRIV, BATV and BUNV.

## Data Availability Statement

The datasets presented in this study can be found in online repositories. The names of the repository/repositories and accession number(s) can be found in the article/Supplementary Material.

## Ethics Statement

Ethical review and approval were not required for the animal study because the samples were taken by Office National de Recherche et de Développement de l’Elevage (ONARDEL) in order to fulfill its governmental mandate to conduct livestock animal monitoring and surveillance programs for veterinary and zoonotic pathogens following all relevant national as well as international regulations and according to fundamental ethical principles. Written informed consent for participation was not obtained from the owners.

## Author Contributions

NC: methodology, practical implementation, data curation and validation, writing of the first draft. YB: conceptualization, practical implementation, data curation. FS: methodology, practical implementation, data curation and validation writing (review and editing). AD: local project implementation. AB: on-site methodology implementation, data curation. UZ, MR, JS, DH: methodology, practical implementation, data curation and validation. MLH: local supervision of project administration. BAD, MYB: resources, project administration. MHG: conceptualization, design of the research strategy and overall supervision of its implementation, data interpretation, funding acquisition and professional project administration, writing. ME: conceptualization, design of the research strategy, methodology, data curation and validation, writing. All authors contributed to the article and approved the submitted version.

## Funding

This work was funded by the German Office for Foreign Affairs (German Partnership Program for Biosecurity, OR12-370-43 BIOS Subsahara). All funding sources were raised by MG. The funders had no role in study design, data collection and analysis, decision to publish, or preparation of the manuscript.

## Conflict of Interest

The authors declare that the research was conducted in the absence of any commercial or financial relationships that could be construed as a potential conflict of interest.

## Publisher’s Note

All claims expressed in this article are solely those of the authors and do not necessarily represent those of their affiliated organizations, or those of the publisher, the editors and the reviewers. Any product that may be evaluated in this article, or claim that may be made by its manufacturer, is not guaranteed or endorsed by the publisher.

## Footnotes

^1^ https://epitools.ausvet.com.au/ciproportion

## Supplementary Material

The Supplementary Material for this article can be found online at: https://www.frontiersin.org/articles/10.3389/fmicb.2021. 766977/full#supplementary-material

Supplementary Figure S1 Alignment of glycoprotein Gc sequences (domains GI and II) of BATV, NRIV and BUNV. Homologous amino acids are marked in color. Alignment is performed with ClystalW.

Supplementary Figure S2 The percentage of NRIV neutralizing antibodies against BATV and NRIV in each individual positive sample. The proportion of NRIV neutralizing antibodies in each sample is indicated in blue and that of BATV neutralizing antibodies in red.
