# Supplementary material for: Co-circulation of Orthobunyaviruses and Rift Valley Fever Virus in Mauritania, 2015
Source: Front Microbiol. 2021 Dec 24;12:766977. doi: 10.3389/fmicb.2021.766977 (PMC8739971; doi:10.3389/fmicb.2021.766977)
Supplement: Supplementary Figure S1 — Alignment of glycoprotein Gc sequences (domains GI and II) of BATV, NRIV and BUNV. Homologous amino acids are marked in color. Alignment is performed with ClystalW. [file Data_Sheet_1.pdf]

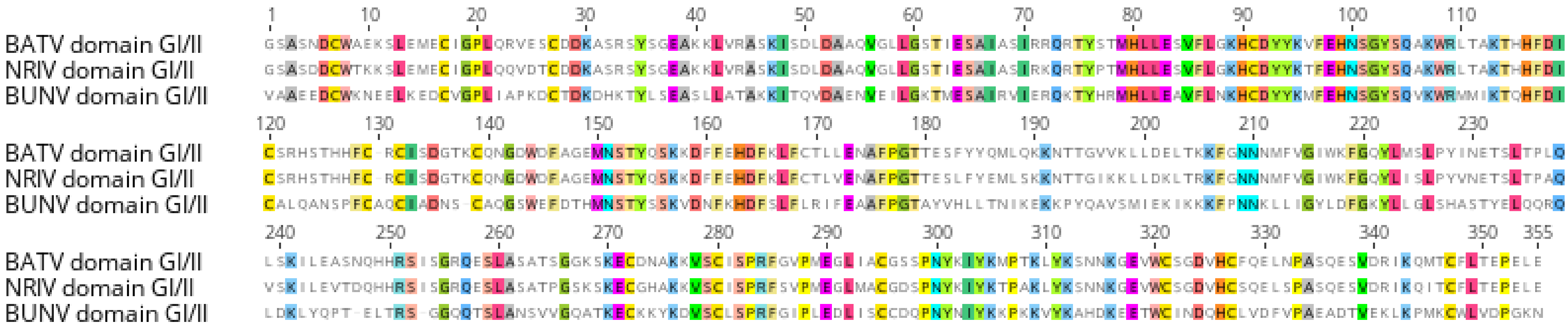
BATV domain GI/II
NRIV domain GI/II
BUNV domain GI/II

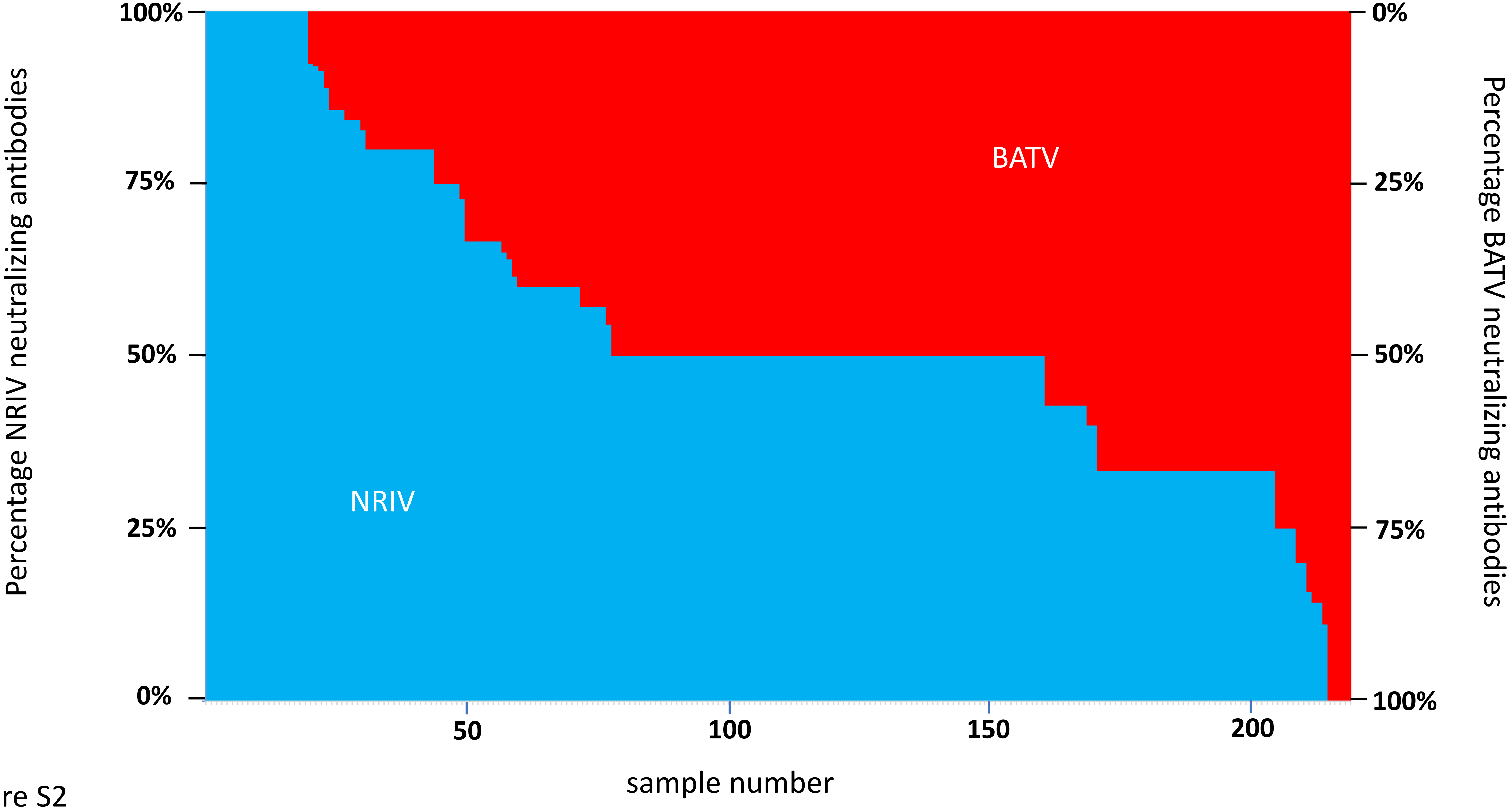
Percentage NRIV neutralizing antibodies
100%
75%
50%
25%
0%
BATV
NRIV
0%
25%
50%
75%
100%
Percentage BATV neutralizing antibodies
50
100
150
200
sample number

Figure S2

Supplemental table| Single values from individual Mauritanian samples tested for antibodies against Nagiri virus (NRIV), Batai virus (BATV) and Bunyamwera virus (BUNV) including virus-antibody-titers from serum neutralisation test (SNT) and ELISA derived optical densities (OD)

numbering	Region			species	sex	age	NRIV SNT	BATV SNT	BUNV SNT	NRIV ELISA [OD_{405}]		mean	OD_{sample}/ OD_{PC}x100	BATV ELISA [OD_{405}]		mean	OD_{sample}/ OD_{PC}x100	BUNV ELISA [OD_{405}]		mean	OD_{sample}/O D_{PC}x100
MR 1 /15 SR	Tasiast	Davass	Mansour Kbeid	cap	m	1	d < 1:10	d < 1:10	d < 1:10	0,15	0,15	0,15	16,24	0,40	0,35	0,37	48,00	0,46	0,44	0,45	31,87
MR 2 /15 SR	Tasiast	Davass	Mansour Kbeid	cap	f	2	d < 1:10	d < 1:10	d < 1:10	0,11	0,10	0,10	11,08	0,20	0,21	0,20	26,19	0,65	0,68	0,67	47,31
MR 3 /15 SR	Tasiast	Davass	Mansour Kbeid	cap	m	3	d < 1:10	d < 1:10	d < 1:10	0,10	0,10	0,10	11,00	0,26	0,26	0,26	33,08	1,03	1,14	1,08	77,11
MR 4 /15 SR	Tasiast	Davass	Mansour Kbeid	cap	f	2	d < 1:10	d < 1:10	d < 1:10	0,09	0,09	0,09	9,45	0,17	0,17	0,17	21,97	0,23	0,22	0,23	16,04
MR 5 /15 SR	Tasiast	Davass	Mansour Kbeid	cap	m	1	d < 1:10	d < 1:10	d < 1:10	0,09	0,10	0,09	9,89	0,32	0,34	0,33	42,69	0,36	0,35	0,36	25,39
MR 6 /15 SR	Tasiast	Davass	Mansour Kbeid	cap	f	1	d < 1:10	d < 1:10	d < 1:10	0,11	0,11	0,11	11,34	0,45	0,45	0,45	57,82	0,35	0,37	0,36	25,49
MR 7 /15 SR	Tasiast	Davass	Mansour Kbeid	cap	f	1	d < 1:10	d < 1:10	d < 1:10	0,09	0,09	0,09	9,21	0,26	0,26	0,26	33,81	0,30	0,31	0,30	21,44
MR 8 /15 SR	Tasiast	Davass	Mansour Kbeid	cap	m	1	d < 1:10	d < 1:10	d < 1:10	0,13	0,15	0,14	14,74	0,44	0,47	0,45	57,93	0,62	0,57	0,60	42,33
MR 9 /15 SR	Tasiast	Davass	Mansour Kbeid	cap	f	2	d < 1:10	d < 1:10	d < 1:10	0,15	0,15	0,15	15,72	0,18	0,19	0,18	23,46	0,44	0,43	0,43	30,85
MR 10 /15 SR	Tasiast	Davass	Mansour Kbeid	cap	m	2	d < 1:10	d < 1:10	d < 1:10	0,13	0,13	0,13	13,57	0,20	0,21	0,20	25,88	0,31	0,30	0,31	21,71
MR 11 /15 SR	Tasiast	Davass	Mansour Kbeid	cap	f	1	d < 1:10	d < 1:10	d < 1:10	0,10	0,09	0,09	10,05	0,18	0,19	0,19	24,26	0,29	0,28	0,28	20,06
MR 12 /15 SR	Tasiast	Davass	Mansour Kbeid	cap	f	1	d < 1:10	d < 1:10	d < 1:10	0,11	0,11	0,11	11,66	0,16	0,17	0,16	21,13	0,39	0,39	0,39	27,58
MR 13 /15 SR	Tasiast	Davass	Mansour Kbeid	cap	m	1	d < 1:10	d < 1:10	d < 1:10	0,16	0,17	0,16	17,45	0,27	0,28	0,28	35,76	0,20	0,20	0,20	14,34
MR 14 /15 SR	Tasiast	Davass	Mansour Kbeid	cap	f	1	d < 1:10	d < 1:10	d < 1:10	0,09	0,09	0,09	6,24	0,18	0,64	0,41	27,76	0,27	0,26	0,26	18,84
MR 15 /15 SR	Tasiast	Davass	Mansour Kbeid	cap	m	2	d < 1:10	d < 1:10	d < 1:10	0,10	0,11	0,11	11,52	0,22	0,22	0,22	28,18	0,41	0,42	0,41	29,47
MR 16 /15 SR	Tasiast	Davass	Melaimine	ov	f	1	d < 1:10	d < 1:10	d < 1:10	0,09	0,09	0,09	9,48	0,13	0,13	0,13	16,47	0,39	0,41	0,40	28,30
MR 17 /15 SR	Tasiast	Davass	Melaimine	ov	m	2	d < 1:10	d < 1:10	d < 1:10	0,11	0,11	0,11	11,52	0,30	0,31	0,31	39,66	0,47	0,48	0,47	33,63
MR 18 /15 SR	Tasiast	Davass	Melaimine	ov	f	1	d < 1:10	d < 1:10	d < 1:10	0,11	0,13	0,12	12,47	0,15	0,15	0,15	19,54	0,18	0,19	0,19	13,19
MR 19 /15 SR	Tasiast	Davass	Melaimine	ov	m	1	d < 1:10	d < 1:10	d < 1:10	0,19	0,18	0,18	19,36	0,22	0,23	0,23	29,05	0,35	0,35	0,35	24,89
MR 20 /15 SR	Tasiast	Davass	Melaimine	ov	f	1	d < 1:10	d < 1:10	d < 1:10	0,08	0,08	0,08	8,70	0,11	0,11	0,11	13,76	0,31	0,34	0,33	23,19
MR 21 /15 SR	Tasiast	Davass	Melaimine	ov	m	2	d < 1:10	d < 1:10	d < 1:10	0,12	0,12	0,12	12,59	0,30	0,31	0,30	38,96	0,83	0,84	0,83	59,26
MR 22 /15 SR	Tasiast	Davass	Melaimine	ov	f	2	d < 1:10	d < 1:10	d < 1:10	0,11	0,13	0,12	12,62	0,59	0,62	0,60	77,47	0,80	0,82	0,81	57,76
MR 23 /15 SR	Tasiast	Davass	Melaimine	ov	f	3	d < 1:10	d < 1:10	d < 1:10	0,10	0,10	0,10	10,68	0,13	0,13	0,13	16,89	0,46	0,46	0,46	32,41
MR 24 /15 SR	Tasiast	Davass	Melaimine	ov	m	1	d < 1:10	d < 1:10	d < 1:10	0,08	0,08	0,08	8,58	0,11	0,11	0,11	14,09	0,34	0,35	0,34	24,46
MR 25 /15 SR	Tasiast	Davass	Melaimine	ov	f	2	d < 1:10	d < 1:10	d < 1:10	0,10	0,10	0,10	10,45	0,37	0,37	0,37	47,68	0,20	0,22	0,21	14,93
MR 26 /15 SR	Tasiast	Davass	Melaimine	ov	m	3	d < 1:10	d < 1:10	d < 1:10	0,11	0,15	0,13	13,82	0,14	0,17	0,15	19,57	0,41	0,43	0,42	29,79
MR 27 /15 SR	Tasiast	Davass	Melaimine	ov	f	2	d < 1:10	d < 1:10	d < 1:10	0,11	0,11	0,11	11,56	0,13	0,17	0,15	19,03	0,17	0,18	0,17	12,42
MR 28 /15 SR	Tasiast	Davass	Melaimine	ov	f	1	d < 1:10	d < 1:10	d < 1:10	0,10	0,09	0,10	10,09	0,11	0,14	0,12	15,86	0,22	0,22	0,22	15,66
MR 29 /15 SR	Tasiast	Davass	Melaimine	ov	f	2	d < 1:10	d < 1:10	d < 1:10	0,09	0,09	0,09	9,43	0,11	0,15	0,13	17,08	0,23	0,23	0,23	16,35
MR 30 /15 SR	Tasiast	Davass	Melaimine	ov	m	1	d < 1:10	d < 1:10	d < 1:10	0,08	0,08	0,08	8,68	0,09	0,12	0,11	14,06	0,26	0,28	0,27	19,17
MR 31 /15 SR	Tasiast	Davass	Melaimine	ov	f	1	d < 1:10	d < 1:10	d < 1:10	0,08	0,08	0,08	8,47	0,09	0,10	0,10	12,29	0,23	0,23	0,23	16,26
MR 32 /15 SR	Tasiast	Davass	Melaimine	ov	m	1	d < 1:10	d < 1:10	d < 1:10	0,08	0,08	0,08	8,58	0,08	0,10	0,09	11,91	0,53	0,54	0,53	38,02
MR 33 /15 SR	Tasiast	Davass	Melaimine	ov	f	2	d < 1:10	d < 1:10	d < 1:10	0,10	0,09	0,09	9,86	0,12	0,14	0,13	16,70	0,15	0,16	0,15	10,90
MR 34 /15 SR	Tasiast	Davass	Melaimine	ov	f	3	d < 1:10	d < 1:10	d < 1:10	0,10	0,14	0,12	12,93	0,15	0,16	0,15	19,55	0,24	0,24	0,24	17,17
MR 35 /15 SR	Tasiast	Davass	Mansour Kbeid	cap	f	3	d < 1:10	d < 1:10	d < 1:10	0,14	0,11	0,13	13,43	0,25	0,26	0,25	32,71	2,00	2,09	2,05	145,52
MR 37 /15 SR	Tasiast	Davass	Mansour Kbeid	cap	f	1	d < 1:10	d < 1:10	d < 1:10	0,14	0,12	0,13	13,55	0,33	0,36	0,35	44,63	0,53	0,52	0,52	37,17
MR 38 /15 SR	Tasiast	Davass	Mansour Kbeid	cap	f	2	d < 1:10	d < 1:10	d < 1:10	0,10	0,10	0,10	10,78	0,17	0,19	0,18	22,77	0,31	0,30	0,30	21,52
MR 39 /15 SR	Tasiast	Davass	Mansour Kbeid	cap	f	3	d < 1:10	d < 1:10	d < 1:10	0,17	0,18	0,18	18,70	0,39	0,43	0,41	52,53	0,72	0,79	0,75	53,58
MR 40 /15 SR	Tasiast	Davass	Mansour Kbeid	cap	f	4	d < 1:10	d < 1:10	d < 1:10	0,46	0,46	0,46	48,42	0,27	0,30	0,29	36,65	0,47	0,52	0,49	35,01
MR 41 /15 SR	Tasiast	Davass	Mansour Kbeid	cap	m	1	d < 1:10	d < 1:10	d < 1:10	0,09	0,09	0,09	9,81	0,17	0,19	0,18	23,18	0,75	0,73	0,74	52,67
MR 42 /15 SR	Tasiast	Davass	Mansour Kbeid	cap	f	2	d < 1:10	d < 1:10	d < 1:10	0,16	0,17	0,16	17,42	0,40	0,39	0,40	51,04	0,61	0,62	0,62	44,02
MR 43 /15 SR	Tasiast	Davass	Mansour Kbeid	cap	f	1	d < 1:10	d < 1:10	d < 1:10	0,30	0,24	0,27	28,86	0,50	0,47	0,49	62,35	0,42	0,43	0,42	30,25
MR 44 /15 SR	Tasiast	Davass	Mansour Kbeid	cap	m	2	d < 1:10	d < 1:10	d < 1:10	0,11	0,12	0,12	12,58	0,24	0,24	0,24	31,11	0,45	0,46	0,45	32,35
MR 45 /15 SR	Tasiast	Davass	Mansour Kbeid	cap	f	1	d < 1:10	d < 1:10	d < 1:10	0,12	0,12	0,12	12,65	0,28	0,32	0,30	38,61	0,35	0,35	0,35	25,22
MR 46 /15 SR	Tasiast	Davass	Mansour Kbeid	cap	m	1	d < 1:10	d < 1:10	d < 1:10	0,24	0,28	0,26	27,79	0,13	0,15	0,14	18,31	0,73	0,74	0,74	52,57
MR 47 /15 SR	Tasiast	Davass	Mansour Kbeid	cap	f	1	d < 1:10	d < 1:10	d < 1:10	0,20	0,20	0,20	21,57	0,49	0,50	0,49	63,48	0,79	0,83	0,81	57,93
MR 48 /15 SR	Tasiast	Davass	Mansour Kbeid	cap	m	2	d < 1:10	d < 1:10	d < 1:10	0,18	0,18	0,18	19,51	0,20	0,21	0,21	26,76	0,73	0,72	0,73	52,05
MR 49 /15 SR	Tasiast	Davass	Mansour Kbeid	cap	f	1	d < 1:10	d < 1:10	d < 1:10	0,10	0,10	0,10	10,87	0,12	0,14	0,13	17,11	0,70	0,71	0,70	50,22
MR 50 /15 SR	Tasiast	Davass	Melaimine	ov	f	2	d < 1:10	d < 1:10	d < 1:10	0,09	0,08	0,08	13,69	0,09	0,09	0,09	6,69	0,17	0,17	0,17	11,98
MR 51 /15 SR	Tasiast	Davass	Melaimine	ov	m	1	d < 1:10	d < 1:10	d < 1:10	0,09	0,08	0,09	14,06	0,09	0,09	0,09	6,68	0,15	0,15	0,15	10,87
MR 52 /15 SR	Tasiast	Davass	Melaimine	ov	f	2	d < 1:10	d < 1:10	d < 1:10	0,11	0,09	0,10	16,06	0,10	0,10	0,10	7,17	0,15	0,15	0,15	10,76
MR 54 /15 SR	Tasiast	Davass	Melaimine	ov	f	4	**d 1:15**	d < 1:10	d < 1:10	0,09	0,24	0,16	26,76	0,10	0,10	0,10	6,89	0,29	0,30	0,30	21,10
MR 55 /15 SR	Tasiast	Davass	Melaimine	ov	f	3	d < 1:10	d < 1:10	d < 1:10	0,09	0,09	0,09	14,55	0,09	0,09	0,09	6,65	0,22	0,22	0,22	16,04
MR 56 /15 SR	Tasiast	Davass	Melaimine	ov	f	4	d < 1:10	d < 1:10	d < 1:10	0,08	0,08	0,08	12,94	0,10	0,10	0,10	7,04	0,13	0,13	0,13	9,28
MR 58 /15 SR	Tasiast	Davass	Melaimine	ov	f	2	d < 1:10	d < 1:10	d < 1:10	0,16	0,15	0,16	25,68	0,10	0,11	0,10	7,42	0,43	0,43	0,43	30,53
MR 59 /15 SR	Tasiast	Davass	Melaimine	ov	m	1	d < 1:10	d < 1:10	d < 1:10	0,10	0,09	0,09	14,89	0,10	0,10	0,10	7,25	0,21	0,21	0,21	14,91

MR 60 /15 SR	Tasiast	Davass	Melaimine	ov	f	1	d < 1:10	d < 1:10	d < 1:10	0,10	0,09	0,09	15,20	0,10	0,10	0,10	7,11	1,34	1,19	1,27	90,47
MR 61 /15 SR	Taziast	n.s.	n.s.	cap	f	4	d < 1:10	d < 1:10	d < 1:10	0,10	0,10	0,10	16,54	0,12	0,12	0,12	8,85	0,23	0,24	0,24	16,94
MR 62 /15 SR	Taziast	n.s.	n.s.	cap	f	3	d < 1:10	d < 1:10	d < 1:10	0,09	0,09	0,09	14,55	0,12	0,13	0,13	8,96	0,16	0,16	0,16	11,25
MR 63 /15 SR	Taziast	n.s.	n.s.	cap	f	2	d < 1:10	d < 1:10	d < 1:10	0,10	0,10	0,10	16,38	0,15	0,14	0,15	10,42	0,26	0,27	0,27	19,12
MR 65 /15 SR	Taziast	n.s.	n.s.	cap	f	3	d < 1:10	d < 1:10	d < 1:10	0,12	0,12	0,12	20,13	0,50	0,51	0,51	36,21	0,84	0,85	0,85	60,62
MR 66 /15 SR	Taziast	n.s.	n.s.	cap	f	3	d < 1:10	d < 1:10	d < 1:10	0,18	0,16	0,17	27,63	0,32	0,33	0,33	23,27	0,89	0,90	0,90	64,15
MR 67 /15 SR	Taziast	n.s.	n.s.	cap	f	2	d < 1:10	d < 1:10	d < 1:10	0,11	0,11	0,11	17,79	0,25	0,25	0,25	18,12	0,41	0,41	0,41	29,23
MR 68 /15 SR	Taziast	n.s.	n.s.	cap	f	3	**d 1:10**	d < 1:10	d < 1:10	0,27	0,23	0,25	40,96	0,51	0,51	0,51	36,27	0,44	0,45	0,44	31,61
MR 69 /15 SR	Taziast	n.s.	n.s.	cap	f	3	d < 1:10	d < 1:10	d < 1:10	0,10	0,10	0,10	16,62	0,17	0,17	0,17	12,05	0,34	0,35	0,34	24,54
MR 70 /15 SR	Taziast	n.s.	n.s.	cap	f	2	d < 1:10	d < 1:10	d < 1:10	0,13	0,14	0,13	21,60	0,20	0,19	0,20	14,13	0,47	0,47	0,47	33,49
MR 71 /15 SR	Taziast	n.s.	n.s.	cap	f	4	d < 1:10	d < 1:10	d < 1:10	0,09	0,10	0,10	15,65	0,11	0,11	0,11	7,77	1,23	1,29	1,26	90,25
MR 72 /15 SR	Taziast	n.s.	n.s.	cap	f	4	d < 1:10	d < 1:10	d < 1:10	0,19	0,18	0,19	30,25	0,28	0,29	0,28	20,02	0,86	0,87	0,86	61,76
MR 73 /15 SR	Taziast	n.s.	n.s.	cap	f	3	d < 1:10	d < 1:10	d < 1:10	0,10	0,11	0,10	16,95	0,23	0,22	0,23	16,38	0,49	0,48	0,49	34,73
MR 74 /15 SR	Taziast	n.s.	n.s.	cap	f	5	d < 1:10	d < 1:10	d < 1:10	0,10	0,10	0,10	16,15	0,15	0,15	0,15	10,50	0,55	0,55	0,55	39,26
MR 75 /15 SR	Taziast	n.s.	n.s.	cap	f	3	d < 1:10	d < 1:10	d < 1:10	0,11	0,11	0,11	17,67	0,19	0,18	0,19	13,18	0,51	0,50	0,50	35,93
MR 76 /15 SR	Taziast	n.s.	n.s.	cap	f	2	d < 1:10	d < 1:10	d < 1:10	0,09	0,09	0,09	14,68	0,11	0,11	0,11	8,07	0,24	0,26	0,25	17,59
MR 77 /15 SR	Taziast	n.s.	n.s.	cap	f	4	d < 1:10	d < 1:10	d < 1:10	0,10	0,10	0,10	16,13	0,13	0,13	0,13	9,45	0,64	0,66	0,65	46,35
MR 78 /15 SR	Taziast	n.s.	n.s.	cap	f	3	**d 1:10**	d < 1:10	d < 1:10	0,09	0,09	0,09	14,60	0,12	0,12	0,12	8,41	0,22	0,24	0,23	16,33
MR 79 /15 SR	Taziast	n.s.	n.s.	cap	f	5	d < 1:10	d < 1:10	d < 1:10	0,10	0,12	0,11	18,18	0,18	0,18	0,18	12,53	0,22	0,22	0,22	15,83
MR 80 /15 SR	Taziast	n.s.	n.s.	cap	f	3	d < 1:10	d < 1:10	d < 1:10	0,08	0,09	0,09	14,08	0,12	0,12	0,12	8,55	0,26	0,29	0,28	19,69
MR 81 /15 SR	Taziast	n.s.	n.s.	cap	f	3	d < 1:10	d < 1:10	d < 1:10	0,08	0,08	0,08	13,05	0,13	0,13	0,13	9,28	0,17	0,17	0,17	12,13
MR 82 /15 SR	Chargui	Bassiknou	n.s.	ov	f	3	d < 1:10	d < 1:10	d < 1:10	0,11	0,12	0,12	18,80	0,20	0,20	0,20	14,30	0,72	0,72	0,72	51,65
MR 83 /15 SR	Chargui	Bassiknou	n.s.	ov	f	5	**d ≥ 1:30**	**d ≥ 1:30**	d < 1:10	0,28	0,27	0,28	45,03	0,68	0,65	0,67	47,41	1,08	1,17	1,12	80,26
MR 84 /15 SR	Chargui	Bassiknou	n.s.	ov	f	4	**d ≥ 1:30**	**d ≥ 1:60**	d < 1:10	0,52	0,50	0,51	83,65	0,57	0,61	0,59	42,15	0,38	0,38	0,38	25,88
MR 85 /15 SR	Chargui	Bassiknou	n.s.	ov	f	3	**d ≥ 1:30**	**d ≥ 1:60**	d 1:10	0,49	0,46	0,47	77,12	0,63	0,59	0,61	43,38	0,77	0,76	0,77	52,68
MR 86 /15 SR	Chargui	Bassiknou	n.s.	ov	f	3	**d ≥ 1:30**	**d ≥ 1:60**	d < 1:10	0,30	0,27	0,28	46,36	0,30	0,32	0,31	22,22	0,18	0,19	0,18	12,72
MR 87 /15 SR	Chargui	Bassiknou	n.s.	ov	f	2	**d 1:20**	**d ≥ 1:60**	d < 1:10	0,48	0,43	0,46	74,43	0,87	0,87	0,87	62,09	0,69	0,71	0,70	48,18
MR 88 /15 SR	Chargui	Bassiknou	n.s.	ov	f	4	d < 1:10	d < 1:10	d < 1:10	0,13	0,13	0,13	20,76	0,23	0,23	0,23	16,53	0,72	0,74	0,73	50,21
MR 89 /15 SR	Chargui	Bassiknou	n.s.	ov	f	2	**d 1:20**	**d ≥ 1:60**	d < 1:10	0,59	0,19	0,39	63,26	0,74	0,73	0,73	52,09	0,73	0,76	0,74	51,14
MR 90 /15 SR	Chargui	Bassiknou	n.s.	ov	f	4	d < 1:10	d < 1:10	d < 1:10	0,11	0,09	0,10	16,15	0,16	0,17	0,17	11,95	0,48	0,49	0,48	33,29
MR 91 /15 SR	Chargui	Bassiknou	n.s.	ov	f	2	**d ≥ 1:30**	**d 1:30**	d 1:15	0,27	0,22	0,24	39,64	0,39	0,42	0,41	28,99	0,85	0,86	0,85	58,76
MR 92 /15 SR	Chargui	Bassiknou	n.s.	ov	f	5	d < 1:10	d < 1:10	d < 1:10	0,13	0,13	0,13	21,27	0,11	0,11	0,11	8,06	0,37	0,38	0,37	25,52
MR 93 /15 SR	Chargui	Bassiknou	n.s.	ov	f	3	d < 1:10	d < 1:10	d < 1:10	0,11	0,11	0,11	17,39	0,13	0,13	0,13	9,52	0,54	0,57	0,56	38,26
MR 94 /15 SR	Chargui	Bassiknou	n.s.	ov	f	2	d < 1:10	d < 1:10	d < 1:10	0,10	0,10	0,10	16,64	0,14	0,14	0,14	10,01	0,83	0,82	0,83	56,78
MR 95 /15 SR	Chargui	Bassiknou	n.s.	ov	f	3	**d 1:10**	d < 1:10	d < 1:10	0,10	0,10	0,10	16,37	0,16	0,16	0,16	11,44	0,23	0,23	0,23	15,73
MR 96 /15 SR	Chargui	Bassiknou	n.s.	ov	f	5	**d 1:20**	**d 1:10**	d < 1:10	0,32	0,31	0,32	51,76	0,52	0,50	0,51	36,98	0,56	0,55	0,55	38,17
MR 97 /15 SR	Chargui	Bassiknou	n.s.	ov	f	3	**d ≥ 1:30**	d < 1:10	d < 1:10	0,13	0,13	0,13	17,00	0,17	0,16	0,16	11,91	0,93	0,91	0,92	63,43
MR 98 /15 SR	Chargui	Bassiknou	n.s.	ov	f	3	**d ≥ 1:30**	**d ≥ 1:30**	d < 1:10	0,42	0,39	0,41	52,42	0,56	0,60	0,58	42,10	0,81	0,82	0,81	55,80
MR 99 /15 SR	Chargui	Bassiknou	n.s.	ov	f	2	d < 1:10	d < 1:10	d < 1:10	0,12	0,11	0,11	14,33	0,14	0,14	0,14	10,01	0,25	0,25	0,25	17,07
MR 100 /15 SR	Chargui	Bassiknou	n.s.	ov	f	1	d < 1:10	d < 1:10	d < 1:10	0,49	0,46	0,47	61,37	0,58	0,53	0,55	40,10	1,34	1,35	1,34	92,43
MR 101 /15 SR	Chargui	Bassiknou	n.s.	ov	f	2	d < 1:10	d < 1:10	d < 1:10	0,10	0,09	0,10	12,38	0,17	0,17	0,17	12,43	0,23	0,23	0,23	15,87
MR 103 /15 SR	Chargui	Bassiknou	n.s.	ov	f	3	d < 1:10	d < 1:10	d < 1:10	0,13	0,12	0,13	16,17	0,19	0,19	0,19	14,01	0,34	0,35	0,34	23,67
MR 104 /15 SR	Chargui	Bassiknou	n.s.	ov	f	1	d < 1:10	d < 1:10	d < 1:10	0,29	0,20	0,24	31,49	0,82	0,80	0,81	58,68	0,40	0,41	0,40	27,63
MR 105 /15 SR	Chargui	Bassiknou	n.s.	ov	f	3	d < 1:10	d < 1:10	d < 1:10	0,11	0,12	0,11	14,82	0,16	0,15	0,15	11,19	0,52	0,54	0,53	36,32
MR 106 /15 SR	Chargui	Bassiknou	n.s.	ov	f	2	**d ≥ 1:30**	d < 1:10	d < 1:10	2,57	2,59	2,58	333,20	3,92	OVER	3,92	284,56	2,13	2,34	2,24	153,84
MR 107 /15 SR	Chargui	Bassiknou	n.s.	ov	m	4	**d ≥ 1:30**	d < 1:10	d 1:20	0,99	0,98	0,99	127,56	0,95	0,94	0,94	68,47	3,23	3,27	3,25	223,65
MR 108 /15 SR	Chargui	Bassiknou	n.s.	ov	f	3	d < 1:10	d < 1:10	d < 1:10	0,14	0,13	0,14	17,45	0,16	0,15	0,16	11,34	0,32	0,32	0,32	22,13
MR 109 /15 SR	Chargui	Bassiknou	n.s.	ov	f	2	d < 1:10	d < 1:10	d < 1:10	0,12	0,12	0,12	15,13	0,16	0,15	0,15	11,21	0,22	0,22	0,22	14,99
MR 110 /15 SR	Chargui	Bassiknou	n.s.	ov	f	1	d < 1:10	d < 1:10	d < 1:10	0,08	0,09	0,08	10,91	0,16	0,16	0,16	11,36	1,10	1,14	1,12	76,79
MR 111 /15 SR	Chargui	Bassiknou	n.s.	ov	f	1	d < 1:10	d < 1:10	d < 1:10	0,15	0,14	0,14	18,23	2,64	2,59	2,62	189,83	2,20	2,35	2,27	156,45
MR 112 /15 SR	Chargui	Bassiknou	n.s.	ov	f	1	**d 1:15**	**d 1:10**	d < 1:10	0,08	0,09	0,08	10,70	0,10	0,10	0,10	7,52	0,53	0,59	0,56	38,35
MR 113 /15 SR	Chargui	Bassiknou	n.s.	ov	f	1	d < 1:10	d < 1:10	d < 1:10	0,12	0,12	0,12	15,31	0,84	0,88	0,86	62,32	0,40	0,41	0,40	27,76
MR 114 /15 SR	Chargui	Bassiknou	n.s.	ov	f	1	**d 1:10**	d < 1:10	d < 1:10	0,10	0,10	0,10	13,35	0,21	0,21	0,21	15,23	0,47	0,49	0,48	32,95
MR 115 /15 SR	Chargui	Bassiknou	n.s.	ov	f	1	**d ≥ 1:30**	**d ≥ 1:60**	d < 1:10	1,45	1,29	1,37	177,19	1,83	1,79	1,81	131,21	0,38	0,40	0,39	26,86
MR 116 /15 SR	Chargui	Bassiknou	n.s.	ov	f	1	d < 1:10	d < 1:10	d < 1:10	0,09	0,09	0,09	12,12	0,13	0,13	0,13	9,61	0,67	0,67	0,67	46,18
MR 117 /15 SR	Chargui	Bassiknou	n.s.	ov	f	3	d < 1:10	d < 1:10	d < 1:10	0,23	0,22	0,22	28,92	1,36	1,33	1,34	97,24	0,68	0,76	0,72	49,37
MR 118 /15 SR	Chargui	Bassiknou	n.s.	ov	f	1	**d 1:15**	d < 1:10	d < 1:10	0,09	0,09	0,09	11,51	0,13	0,13	0,13	9,55	0,17	0,22	0,20	13,53
MR 119 /15 SR	Chargui	Bassiknou	n.s.	ov	f	2	d < 1:10	d < 1:10	d < 1:10	0,08	0,08	0,08	10,16	0,10	0,10	0,10	7,50	0,21	0,22	0,22	14,90
MR 120 /15 SR	Chargui	Bassiknou	n.s.	ov	f	2	**d ≥ 1:30**	**d 1:20**	d < 1:10	0,13	0,12	0,12	16,01	0,27	0,29	0,28	20,47	0,39	0,38	0,39	26,74
MR 121 /15 SR	Chargui	Bassiknou	n.s.	ov	f	3	d < 1:10	d < 1:10	d < 1:10	0,37	0,33	0,35	45,67	0,33	0,33	0,33	23,93	0,42	0,43	0,43	29,30
MR 122 /15 SR	Chargui	Bassiknou	n.s.	cap	f	4	**d ≥ 1:30**	**d ≥ 1:30**	d 1:15	0,49	0,40	0,45	57,66	0,76	0,76	0,76	54,86	0,63	0,65	0,64	44,33
MR 123 /15 SR	Chargui	Bassiknou	n.s.	cap	f	3	**d ≥ 1:30**	**d ≥ 1:30**	d < 1:10	0,33	0,29	0,31	40,58	0,53	0,53	0,53	38,52	1,24	1,31	1,27	87,68

**MR 124 /15 SR**	Chargui	Bassiknou	n.s.	cap	f	7	**d ≥ 1:30**	**d ≥ 1:30**	d 1:20	0,62	0,60	0,61	79,09	0,98	0,98	0,98	71,09	0,58	0,68	0,63	43,31
**MR 125 /15 SR**	Chargui	Bassiknou	n.s.	cap	f	4	**d ≥ 1:30**	**d ≥ 1:30**	d < 1:10	0,47	0,44	0,45	58,56	0,58	0,56	0,57	41,14	0,95	0,98	0,97	68,48
**MR 126 /15 SR**	Chargui	Bassiknou	n.s.	cap	f	5	**d ≥ 1:30**	**d ≥ 1:30**	d 1:15	0,78	0,46	0,62	79,99	1,19	1,14	1,17	84,69	0,69	0,74	0,72	50,73
**MR 127 /15 SR**	Chargui	Bassiknou	n.s.	cap	f	6	**d ≥ 1:30**	**d ≥ 1:30**	d 1:10	0,32	0,27	0,30	38,25	0,53	0,54	0,54	38,91	0,65	0,67	0,66	46,51
**MR 128 /15 SR**	Chargui	Bassiknou	n.s.	cap	f	3	**d ≥ 1:30**	**d ≥ 1:30**	d 1:10	2,49	2,30	2,39	308,90	3,72	3,53	3,63	262,99	2,73	2,91	2,82	199,42
**MR 129 /15 SR**	Chargui	Bassiknou	n.s.	cap	f	8	**d ≥ 1:30**	**d ≥ 1:30**	d < 1:10	0,20	0,18	0,19	24,86	0,47	0,48	0,48	34,65	0,88	0,88	0,88	62,24
**MR 130 /15 SR**	Chargui	Bassiknou	n.s.	cap	f	1	**d ≥ 1:30**	**d ≥ 1:60**	d 1:10	2,09	2,10	2,10	271,07	2,34	2,26	2,30	166,74	0,41	0,45	0,43	30,23
**MR 131 /15 SR**	Chargui	Bassiknou	n.s.	cap	f	1	d < 1:10	d < 1:10	d < 1:10	0,48	0,40	0,44	57,04	0,44	0,46	0,45	32,70	0,50	0,50	0,50	35,23
**MR 132 /15 SR**	Chargui	Bassiknou	n.s.	cap	f	8	**d ≥ 1:30**	**d ≥ 1:60**	d < 1:10	2,85	2,84	2,85	367,89	2,20	2,24	2,22	148,73	0,69	0,71	0,70	49,47
**MR 133 /15 SR**	Chargui	Bassiknou	n.s.	cap	f	3	**d ≥ 1:30**	**d ≥ 1:60**	d < 1:10	1,61	1,42	1,51	195,69	2,06	1,98	2,02	135,19	3,96	OVER	3,96	279,83
**MR 134 /15 SR**	Chargui	Bassiknou	n.s.	cap	f	3	**d ≥ 1:30**	**d ≥ 1:60**	d 1:10	0,81	0,73	0,77	99,46	1,99	1,95	1,97	131,76	1,40	1,49	1,45	102,23
**MR 135 /15 SR**	Chargui	Bassiknou	n.s.	cap	f	4	**d ≥ 1:30**	**d ≥ 1:60**	d < 1:10	0,36	0,33	0,34	44,51	0,55	0,54	0,55	36,74	0,60	0,59	0,60	42,21
**MR 136 /15 SR**	Chargui	Bassiknou	n.s.	cap	f	3	**d ≥ 1:30**	**d ≥ 1:60**	d 1:10	0,90	0,80	0,85	109,35	1,25	1,20	1,23	82,28	2,69	2,71	2,70	191,01
**MR 137 /15 SR**	Chargui	Bassiknou	n.s.	cap	f	4	**d ≥ 1:30**	**d ≥ 1:60**	d 1:15	0,89	0,95	0,92	119,09	0,72	0,65	0,68	45,75	0,89	0,88	0,88	62,58
**MR 138 /15 SR**	Chargui	Bassiknou	n.s.	cap	f	6	**d ≥ 1:30**	**d ≥ 1:30**	d < 1:10	0,55	0,57	0,56	72,53	0,63	0,58	0,60	40,44	1,05	1,13	1,09	77,12
**MR 139 /15 SR**	Chargui	Bassiknou	n.s.	cap	f	3	**d ≥ 1:30**	**d ≥ 1:30**	d < 1:10	1,05	1,00	1,02	132,26	1,49	1,39	1,44	96,43	0,92	0,92	0,92	64,88
**MR 140 /15 SR**	Chargui	Bassiknou	n.s.	cap	f	3	**d ≥ 1:30**	**d ≥ 1:30**	d < 1:10	1,52	1,25	1,39	179,21	1,63	1,62	1,63	108,83	0,56	0,58	0,57	40,45
**MR 141 /15 SR**	Chargui	Bassiknou	n.s.	cap	f	4	**d ≥ 1:30**	**d ≥ 1:30**	d 1:15	0,33	0,38	0,36	46,21	0,34	0,34	0,34	22,79	0,85	0,84	0,85	59,85
**MR 142 /15 SR**	Chargui	Bassiknou	n.s.	cap	f	3	d < 1:10	**d ≥ 1:30**	d 1:15	0,31	0,27	0,29	37,85	0,42	0,40	0,41	27,49	0,49	0,50	0,49	34,99
**MR 143 /15 SR**	Chargui	Bassiknou	n.s.	cap	f	10	**d ≥ 1:30**	**d ≥ 1:30**	d 1:10	0,96	0,84	0,90	117,75	1,49	1,47	1,48	99,11	1,88	1,93	1,91	134,89
**MR 144 /15 SR**	Chargui	Bassiknou	n.s.	cap	f	3	**d ≥ 1:30**	**d ≥ 1:30**	d 1:10	0,24	0,21	0,23	29,69	0,33	0,32	0,33	21,89	0,67	0,69	0,68	47,84
**MR 145 /15 SR**	Chargui	Bassiknou	n.s.	cap	f	7	**d ≥ 1:30**	**d ≥ 1:30**	d < 1:10	0,42	0,42	0,42	54,70	0,50	0,49	0,50	33,24	0,76	0,78	0,77	54,56
**MR 146 /15 SR**	Chargui	Bassiknou	n.s.	cap	f	5	**d ≥ 1:30**	**d ≥ 1:30**	d < 1:10	1,55	1,43	1,49	194,49	2,00	1,88	1,94	129,91	0,61	0,62	0,62	43,59
**MR 147 /15 SR**	Chargui	Bassiknou	n.s.	cap	f	7	**d ≥ 1:30**	**d ≥ 1:30**	d < 1:10	0,74	0,71	0,72	94,49	1,46	1,43	1,45	96,79	1,20	1,12	1,16	81,99
**MR 148 /15 SR**	Chargui	Bassiknou	n.s.	cap	f	1	d < 1:10	**d 1:10**	d < 1:10	0,17	0,17	0,17	22,26	0,35	0,35	0,35	23,71	0,78	0,82	0,80	56,47
**MR 149 /15 SR**	Chargui	Bassiknou	n.s.	cap	f	6	**d ≥ 1:30**	**d ≥ 1:30**	d < 1:10	0,50	0,47	0,48	63,30	0,56	0,55	0,56	37,26	0,41	0,42	0,41	29,05
**MR 150 /15 SR**	Chargui	Bassiknou	n.s.	cap	m	2	**d ≥ 1:30**	**d ≥ 1:30**	d 1:20	1,77	1,89	1,83	238,90	2,98	2,90	2,94	196,83	0,70	0,74	0,72	51,03
**MR 151 /15 SR**	Chargui	Bassiknou	n.s.	cap	f	2	d < 1:10	**d 1:10**	d < 1:10	0,11	0,11	0,11	14,39	0,14	0,14	0,14	9,57	1,24	1,27	1,26	88,92
**MR 152 /15 SR**	Chargui	Bassiknou	n.s.	cap	f	4	**d ≥ 1:30**	**d ≥ 1:30**	d 1:20	1,05	1,05	1,05	137,53	1,79	1,67	1,73	115,89	1,25	1,27	1,26	88,97
**MR 153 /15 SR**	Chargui	Bassiknou	n.s.	cap	f	5	**d ≥ 1:30**	**d ≥ 1:30**	d < 1:10	0,76	0,72	0,74	96,73	1,53	1,49	1,51	101,23	2,26	2,33	2,29	162,26
**MR 154 /15 SR**	Chargui	Bassiknou	n.s.	cap	f	6	**d ≥ 1:30**	**d ≥ 1:60**	d 1:20	1,73	1,54	1,63	213,38	2,48	2,47	2,48	165,98	0,38	0,39	0,39	27,27
**MR 155 /15 SR**	Chargui	Bassiknou	n.s.	cap	f	4	**d ≥ 1:30**	**d ≥ 1:60**	d 1:20	0,94	0,96	0,95	123,80	2,04	1,77	1,91	127,74	1,35	1,45	1,40	98,78
**MR 156 /15 SR**	Chargui	Bassiknou	n.s.	cap	f	4	d < 1:10	d < 1:10	d < 1:10	0,10	0,10	0,10	12,75	0,16	0,15	0,15	10,31	0,29	0,28	0,29	20,35
**MR 157 /15 SR**	Chargui	Bassiknou	n.s.	cap	f	6	**d 1:10**	d < 1:10	d < 1:10	0,16	0,16	0,16	20,67	0,25	0,25	0,25	16,73	3,10	3,31	3,20	226,61
**MR 158 /15 SR**	Chargui	Bassiknou	n.s.	cap	f	7	d < 1:10	d < 1:10	d < 1:10	0,12	0,12	0,12	15,67	0,21	0,21	0,21	13,98	0,41	0,44	0,42	29,74
**MR 159 /15 SR**	Chargui	Bassiknou	n.s.	cap	f	8	**d ≥ 1:30**	**d ≥ 1:60**	d < 1:10	0,33	0,31	0,32	41,54	0,43	0,41	0,42	27,98	0,56	0,60	0,58	40,83
**MR 160 /15 SR**	Chargui	Bassiknou	n.s.	cap	f	6	d < 1:10	d < 1:10	d < 1:10	0,11	0,11	0,11	13,90	0,22	0,22	0,22	14,76	0,81	0,79	0,80	56,63
**MR 161 /15 SR**	Chargui	Bassiknou	n.s.	cap	f	5	d < 1:10	d < 1:10	d < 1:10	0,08	0,08	0,08	10,69	0,12	0,12	0,12	8,05	0,64	0,70	0,67	47,15
**MR 162 /15 SR**	Chargui	Bassiknou	n.s.	cap	f	5	**d ≥ 1:30**	**d ≥ 1:30**	d 1:10	0,55	0,48	0,52	67,57	0,73	0,73	0,73	48,56	0,58	0,58	0,58	41,05
**MR 163 /15 SR**	Garbie	n.s.	n.s.	ov	f	2	**d ≥ 1:30**	**d ≥ 1:30**	**d ≥ 1:60**	0,66	0,54	0,60	78,64	0,34	0,32	0,33	22,06	0,49	0,49	0,49	34,69
**MR 164 /15 SR**	Garbie	n.s.	n.s.	ov	f	2	d < 1:10	d < 1:10	d < 1:10	0,15	0,14	0,15	19,40	0,21	0,26	0,24	15,75	1,57	1,67	1,62	114,43
**MR 165 /15 SR**	Garbie	n.s.	n.s.	ov	f	5	d < 1:10	d < 1:10	d < 1:10	0,25	0,24	0,24	31,97	0,26	0,24	0,25	16,73	0,67	0,66	0,66	50,55
**MR 166 /15 SR**	Garbie	n.s.	n.s.	ov	f	2	d < 1:10	d < 1:10	d < 1:10	0,09	0,08	0,09	11,34	0,13	0,13	0,13	8,48	0,44	0,45	0,44	33,84
**MR 167 /15 SR**	Garbie	n.s.	n.s.	ov	f	2	**d ≥ 1:30**	**d ≥ 1:30**	d < 1:10	0,70	0,70	0,70	91,34	0,93	0,91	0,92	61,42	0,41	0,41	0,41	31,41
**MR 168 /15 SR**	Garbie	n.s.	n.s.	ov	f	4	d < 1:10	d < 1:10	d < 1:10	0,10	0,10	0,10	12,62	0,11	0,11	0,11	7,46	0,27	0,27	0,27	20,69
**MR 169 /15 SR**	Garbie	n.s.	n.s.	ov	f	2	d < 1:10	d < 1:10	d 1:10	0,19	0,19	0,19	24,56	0,31	0,29	0,30	20,01	0,38	0,38	0,38	28,91
**MR 170 /15 SR**	Garbie	n.s.	n.s.	ov	f	2	**d ≥ 1:30**	**d ≥ 1:30**	d < 1:10	0,15	0,15	0,15	19,17	0,18	0,17	0,17	11,70	0,67	0,71	0,69	52,72
**MR 171 /15 SR**	Garbie	n.s.	n.s.	ov	f	4	d < 1:10	d < 1:10	d < 1:10	0,10	0,10	0,10	13,03	0,17	0,23	0,20	13,24	0,22	0,22	0,22	17,17
**MR 172 /15 SR**	Garbie	n.s.	n.s.	ov	f	1	**d 1:10**	d < 1:10	d < 1:10	0,21	0,20	0,21	26,93	0,26	0,27	0,26	17,95	0,74	0,73	0,74	56,27
**MR 173 /15 SR**	Garbie	n.s.	n.s.	ov	f	5	d < 1:10	d < 1:10	d < 1:10	0,11	0,12	0,11	14,94	0,16	0,16	0,16	11,04	0,39	0,37	0,38	29,01
**MR 174 /15 SR**	Garbie	n.s.	n.s.	ov	f	6	d < 1:10	d < 1:10	d < 1:10	0,11	0,10	0,11	13,75	0,19	0,20	0,20	13,44	0,52	0,56	0,54	41,25
**MR 175 /15 SR**	Garbie	n.s.	n.s.	ov	f	6	**d ≥ 1:30**	**d ≥ 1:30**	d < 1:10	0,14	0,14	0,14	17,91	0,19	0,19	0,19	12,80	0,22	0,22	0,22	16,92
**MR 176 /15 SR**	Garbie	n.s.	n.s.	ov	f	3	d < 1:10	d < 1:10	d < 1:10	0,09	0,08	0,09	11,15	0,11	0,11	0,11	7,61	0,26	0,27	0,26	20,19
**MR 177 /15 SR**	Garbie	n.s.	n.s.	ov	f	2	d < 1:10	d < 1:10	d < 1:10	0,10	0,10	0,10	13,37	0,13	0,12	0,12	8,56	0,39	0,37	0,38	28,97
**MR 178 /15 SR**	Garbie	n.s.	n.s.	ov	f	2	d < 1:10	d < 1:10	d < 1:10	0,11	0,11	0,11	13,98	0,23	0,24	0,24	16,16	0,92	1,00	0,96	72,99
**MR 179 /15 SR**	Garbie	n.s.	n.s.	ov	f	3	**d 1:20**	**d ≥ 1:60**	d 1:10	0,24	0,22	0,23	29,55	0,40	0,41	0,41	27,99	0,64	0,66	0,65	49,69
**MR 180 /15 SR**	Garbie	n.s.	n.s.	ov	f	3	**d 1:15**	d < 1:10	d < 1:10	0,14	0,13	0,13	17,40	0,28	0,26	0,27	18,46	0,29	0,30	0,29	22,38
**MR 181 /15 SR**	Garbie	n.s.	n.s.	ov	f	2	d < 1:10	d < 1:10	d < 1:10	0,09	0,10	0,10	12,43	0,11	0,11	0,11	7,71	0,18	0,19	0,18	14,09
**MR 182 /15 SR**	Garbie	n.s.	n.s.	ov	f	3	**d ≥ 1:30**	**d 1:40**	d < 1:10	0,08	0,09	0,08	11,04	0,10	0,10	0,10	6,65	1,27	1,32	1,30	99,05
**MR 183 /15 SR**	Garbie	n.s.	n.s.	ov	f	1	**d ≥ 1:30**	**d ≥ 1:60**	d 1:30	0,97	0,86	0,91	119,45	1,31	1,31	1,31	89,95	1,53	1,51	1,52	116,04
**MR 184 /15 SR**	Garbie	n.s.	n.s.	ov	f	7	**d ≥ 1:30**	**d ≥ 1:30**	d < 1:10	0,39	0,32	0,35	46,26	0,57	0,55	0,56	38,53	0,21	0,21	0,21	16,05
**MR 185 /15 SR**	Garbie	n.s.	n.s.	ov	f	2	**d ≥ 1:30**	d < 1:10	d < 1:10	0,10	0,09	0,10	12,53	0,11	0,11	0,11	7,84	0,19	0,20	0,19	14,75

**MR 186 /15 SR**	Garbie	n.s.	n.s.	ov	f	5	d < 1:10	d < 1:10	d < 1:10	0,10	0,09	0,10	12,45	0,11	0,11	0,11	7,46	0,22	0,22	0,22	16,84
**MR 187 /15 SR**	Garbie	n.s.	n.s.	ov	f	2	**d ≥ 1:30**	**d ≥ 1:30**	d 1:15	0,75	0,70	0,72	100,45	1,06	1,03	1,05	71,89	0,39	0,37	0,38	29,04
**MR 188 /15 SR**	Garbie	n.s.	n.s.	ov	f	4	d < 1:10	d < 1:10	d < 1:10	0,20	0,20	0,20	27,49	0,38	0,37	0,37	25,44	0,31	0,31	0,31	23,72
**MR 189 /15 SR**	Garbie	n.s.	n.s.	ov	f	1	d < 1:10	d < 1:10	d < 1:10	0,11	0,10	0,10	14,15	0,15	0,15	0,15	10,41	0,32	0,32	0,32	24,38
**MR 190 /15 SR**	Garbie	n.s.	n.s.	ov	f	6	**d ≥ 1:30**	**d ≥ 1:30**	d < 1:10	0,33	0,31	0,32	44,34	0,51	0,51	0,51	35,12	0,39	0,40	0,39	29,83
**MR 191 /15 SR**	Garbie	n.s.	n.s.	ov	f	5	d < 1:10	d < 1:10	d < 1:10	0,11	0,10	0,11	15,03	0,14	0,14	0,14	9,43	0,35	0,36	0,36	27,30
**MR 192 /15 SR**	Garbie	n.s.	n.s.	ov	f	5	**d ≥ 1:30**	**d ≥ 1:30**	d < 1:10	0,66	0,58	0,62	86,19	0,46	0,45	0,46	31,27	0,38	0,39	0,38	29,30
**MR 193 /15 SR**	Garbie	n.s.	n.s.	ov	f	6	**d ≥ 1:30**	**d ≥ 1:30**	d 1:10	0,29	0,26	0,27	37,99	0,36	0,36	0,36	24,73	0,37	0,36	0,36	27,77
**MR 194 /15 SR**	Garbie	n.s.	n.s.	ov	f	6	**d ≥ 1:30**	**d ≥ 1:30**	d 1:15	0,58	0,51	0,54	75,23	0,68	0,70	0,69	47,17	0,27	0,29	0,28	21,33
**MR 195 /15 SR**	Garbie	n.s.	n.s.	ov	f	5	d < 1:10	d < 1:10	d < 1:10	0,08	0,08	0,08	10,96	0,09	0,09	0,09	6,15	0,30	0,30	0,30	22,84
**MR 196 /15 SR**	Garbie	n.s.	n.s.	ov	f	1	d < 1:10	d < 1:10	d < 1:10	0,19	0,19	0,19	26,02	0,31	0,31	0,31	21,28	0,50	0,51	0,50	38,36
**MR 197 /15 SR**	Garbie	n.s.	n.s.	ov	f	2	d < 1:10	d < 1:10	d < 1:10	0,09	0,08	0,08	11,58	0,09	0,09	0,09	6,46	0,46	0,49	0,48	36,58
**MR 198 /15 SR**	Garbie	n.s.	n.s.	ov	f	2	**d 1:10**	d < 1:10	d < 1:10	0,09	0,09	0,09	12,18	0,12	0,12	0,12	8,35	0,85	0,85	0,85	65,14
**MR 200 /15 SR**	Garbie	n.s.	n.s.	ov	f	5	d < 1:10	d < 1:10	d < 1:10	0,08	0,08	0,08	11,39	0,10	0,10	0,10	6,63	0,39	0,37	0,38	29,22
**MR 201 /15 SR**	Garbie	n.s.	n.s.	ov	f	5	**d ≥ 1:30**	**d ≥ 1:60**	d < 1:10	0,66	0,61	0,64	88,14	0,54	0,55	0,54	37,30	0,70	0,67	0,69	52,65
**MR 202 /15 SR**	Garbie	n.s.	n.s.	ov	f	6	**d ≥ 1:30**	**d ≥ 1:60**	d 1:15	1,11	1,11	1,11	153,69	1,10	1,16	1,13	77,48	0,29	0,29	0,29	22,14
**MR 203 /15 SR**	Garbie	n.s.	n.s.	ov	f	2	d < 1:10	d < 1:10	d < 1:10	0,11	0,11	0,11	15,21	0,24	0,24	0,24	16,63	0,17	0,17	0,17	13,09
**MR 204 /15 SR**	Garbie	n.s.	n.s.	ov	f	2	**d 1:10**	d < 1:10	d 1:40	0,09	0,10	0,10	13,19	0,13	0,13	0,13	8,85	2,04	2,11	2,08	158,42
**MR 205 /15 SR**	Garbie	n.s.	n.s.	ov	f	4	**d ≥ 1:30**	**d ≥ 1:60**	d < 1:10	0,40	0,37	0,39	53,88	0,64	0,67	0,65	44,95	2,57	2,55	2,56	197,18
**MR 206 /15 SR**	Garbie	n.s.	n.s.	ov	f	2	d < 1:10	d < 1:10	d < 1:10	0,14	0,13	0,13	18,50	0,22	0,20	0,21	14,44	0,42	0,42	0,42	32,12
**MR 207 /15 SR**	Garbie	n.s.	n.s.	ov	f	5	**d ≥ 1:30**	**d ≥ 1:60**	d 1:10	0,48	0,43	0,46	63,27	0,56	0,53	0,54	37,23	0,29	0,29	0,29	22,25
**MR 208 /15 SR**	Garbie	n.s.	n.s.	ov	f	3	**d ≥ 1:30**	**d ≥ 1:60**	d 1:10	0,27	0,25	0,26	36,15	0,43	0,42	0,43	29,21	0,36	0,37	0,36	27,99
**MR 209 /15 SR**	Garbie	n.s.	n.s.	ov	f	2	d < 1:10	d < 1:10	d < 1:10	0,09	0,09	0,09	12,44	0,11	0,11	0,11	7,42	0,42	0,42	0,42	32,22
**MR 210 /15 SR**	Garbie	n.s.	n.s.	ov	f	5	**d ≥ 1:30**	**d ≥ 1:30**	d < 1:10	0,19	0,17	0,18	25,32	0,18	0,19	0,19	12,78	0,68	0,68	0,68	52,06
**MR 211 /15 SR**	Garbie	n.s.	n.s.	ov	f	6	**d ≥ 1:30**	**d 1:20**	d < 1:10	0,38	0,36	0,37	51,28	0,46	0,45	0,46	31,40	0,33	0,32	0,32	24,88
**MR 212 /15 SR**	Garbie	n.s.	n.s.	ov	f	2	d < 1:10	d < 1:10	d < 1:10	0,10	0,10	0,10	14,12	0,11	0,15	0,13	8,93	0,24	0,24	0,24	18,48
**MR 213 /15 SR**	Garbie	n.s.	n.s.	ov	f	5	d < 1:10	d < 1:10	d < 1:10	0,13	0,13	0,13	17,46	0,18	0,16	0,17	15,45	0,19	0,19	0,19	14,75
**MR 214 /15 SR**	Garbie	n.s.	n.s.	ov	f	2	d < 1:10	d < 1:10	d < 1:10	0,16	0,16	0,16	22,32	0,19	0,18	0,18	16,94	0,28	0,28	0,28	21,89
**MR 215 /15 SR**	Garbie	n.s.	n.s.	ov	f	5	**d ≥ 1:30**	**d ≥ 1:30**	d < 1:10	0,35	0,35	0,35	48,44	0,49	0,47	0,48	44,09	0,50	0,51	0,51	38,95
**MR 216 /15 SR**	Garbie	n.s.	n.s.	ov	f	6	d < 1:10	d < 1:10	d < 1:10	0,20	0,20	0,20	27,78	0,25	0,25	0,25	23,14	0,32	0,31	0,32	24,41
**MR 217 /15 SR**	Garbie	n.s.	n.s.	ov	f	2	d < 1:10	d < 1:10	d < 1:10	0,11	0,11	0,11	14,93	0,13	0,13	0,13	11,65	0,38	0,37	0,37	28,81
**MR 218 /15 SR**	Garbie	n.s.	n.s.	ov	f	6	**d ≥ 1:30**	**d ≥ 1:30**	d 1:10	0,39	0,34	0,36	50,36	0,59	0,59	0,59	54,31	0,54	0,55	0,54	41,92
**MR 219 /15 SR**	Garbie	n.s.	n.s.	ov	f	5	d < 1:10	d < 1:10	d < 1:10	0,12	0,12	0,12	16,74	0,19	0,17	0,18	16,60	0,86	0,85	0,86	65,92
**MR 220 /15 SR**	Garbie	n.s.	n.s.	ov	f	6	**d ≥ 1:30**	**d ≥ 1:30**	d < 1:10	0,13	0,12	0,13	17,35	0,19	0,18	0,18	16,98	0,18	0,17	0,17	13,37
**MR 221 /15 SR**	Garbie	n.s.	n.s.	ov	f	5	d < 1:10	d < 1:10	d < 1:10	0,18	0,17	0,18	24,61	0,33	0,31	0,32	29,34	0,90	0,91	0,91	69,91
**MR 222 /15 SR**	Garbie	n.s.	n.s.	ov	f	5	**d 1:10**	d < 1:10	d < 1:10	0,08	0,08	0,08	10,90	0,11	0,10	0,10	9,60	0,26	0,27	0,26	20,39
**MR 223 /15 SR**	Garbie	n.s.	n.s.	ov	f	3	**d 1:240**	**d 1:80**	**d 1:20**	0,91	0,59	0,75	53,54	2,51	2,06	2,28	154,56	1,17	1,24	1,21	92,92
**MR 224 /15 SR**	Garbie	n.s.	n.s.	ov	f	5	d < 1:10	d < 1:10	d < 1:10	0,09	0,09	0,09	12,81	0,10	0,10	0,10	8,86	0,34	0,36	0,35	26,92
**MR 225 /15 SR**	Garbie	n.s.	n.s.	ov	f	2	d < 1:10	d < 1:10	d < 1:10	0,12	0,13	0,13	17,59	0,24	0,24	0,24	22,31	0,29	0,29	0,29	22,06
**MR 226 /15 SR**	Garbie	n.s.	n.s.	ov	f	2	d < 1:10	d < 1:10	d < 1:10	0,10	0,10	0,10	13,80	0,23	0,23	0,23	21,29	0,95	0,95	0,95	72,92
**MR 227 /15 SR**	Garbie	n.s.	n.s.	ov	f	2	d < 1:10	d < 1:10	d < 1:10	0,09	0,10	0,10	13,20	0,14	0,14	0,14	12,99	0,60	0,58	0,59	45,42
**MR 228 /15 SR**	Garbie	n.s.	n.s.	ov	f	6	d < 1:10	d < 1:10	d < 1:10	0,79	0,71	0,75	104,30	1,08	1,12	1,10	101,69	0,60	0,58	0,59	45,66
**MR 229 /15 SR**	Garbie	n.s.	n.s.	ov	f	2	d < 1:10	d < 1:10	d < 1:10	0,38	0,33	0,35	49,15	1,46	1,39	1,43	131,62	1,61	1,67	1,64	126,29
**MR 230 /15 SR**	Garbie	n.s.	n.s.	ov	f	2	d < 1:10	d < 1:10	d < 1:10	0,17	0,14	0,15	20,85	0,16	0,15	0,15	14,13	0,74	0,74	0,74	56,98
**MR 231 /15 SR**	Garbie	n.s.	n.s.	ov	f	2	d < 1:10	d < 1:10	d < 1:10	0,28	0,28	0,28	38,83	0,38	0,37	0,38	34,80	1,91	1,95	1,93	148,56
**MR 232 /15 SR**	Garbie	n.s.	n.s.	ov	f	2	d < 1:10	d < 1:10	d < 1:10	0,08	0,08	0,08	11,19	0,11	0,11	0,11	9,90	0,51	0,48	0,49	38,12
**MR 233 /15 SR**	Garbie	n.s.	n.s.	ov	f	1	d < 1:10	d < 1:10	d < 1:10	0,18	0,17	0,18	24,50	0,24	0,24	0,24	21,88	1,14	1,16	1,15	88,48
**MR 234 /15 SR**	Garbie	n.s.	n.s.	ov	f	3	d < 1:10	d < 1:10	d < 1:10	0,10	0,10	0,10	13,86	0,10	0,10	0,10	9,59	0,22	0,23	0,23	17,41
**MR 235 /15 SR**	Garbie	n.s.	n.s.	ov	f	6	**d 1:20**	**d ≥ 1:30**	d < 1:10	0,36	0,30	0,33	46,44	0,36	0,36	0,36	33,20	1,06	1,04	1,05	80,70
**MR 236 /15 SR**	Garbie	n.s.	n.s.	ov	f	6	**d ≥ 1:120**	**d 1:120**	d < 1:10	0,86	0,84	0,85	118,06	0,89	0,89	0,89	72,12	0,57	0,57	0,57	44,11
**MR 237 /15 SR**	Garbie	n.s.	n.s.	ov	f	6	**d ≥ 1:30**	**d ≥ 1:30**	d 1:10	0,16	0,12	0,14	19,08	0,34	0,33	0,34	30,91	0,34	0,35	0,35	26,66
**MR 238 /15 SR**	Garbie	n.s.	n.s.	ov	f	2	d < 1:10	d < 1:10	d < 1:10	0,10	0,09	0,10	13,45	0,18	0,18	0,18	16,88	2,16	2,21	2,18	168,07
**MR 239 /15 SR**	Garbie	n.s.	n.s.	ov	f	6	**d ≥ 1:30**	**d 1:20**	d < 1:10	0,13	0,12	0,13	17,72	0,23	0,23	0,23	21,58	0,41	0,40	0,41	31,37
**MR 240 /15 SR**	Garbie	n.s.	n.s.	ov	f	4	d < 1:10	d < 1:10	d < 1:10	0,29	0,28	0,29	40,23	0,28	0,28	0,28	25,91	0,37	0,39	0,38	29,23
**MR 241 /15 SR**	Garbie	n.s.	n.s.	ov	f	4	d < 1:10	d < 1:10	d < 1:10	0,10	0,10	0,10	14,57	0,13	0,13	0,13	12,30	0,56	0,56	0,56	43,20
**MR 242 /15 SR**	Garbie	n.s.	n.s.	ov	m	1	d < 1:10	d < 1:10	d < 1:10	0,09	0,09	0,09	12,97	0,12	0,12	0,12	10,89	0,40	0,33	0,36	28,09
**MR 243 /15 SR**	Garbie	n.s.	n.s.	ov	f	3	d < 1:10	d < 1:10	d < 1:10	0,28	0,28	0,28	39,21	0,43	0,44	0,43	39,88	1,16	1,18	1,17	90,34
**MR 244 /15 SR**	Tagant	Letvetar	n.s.	cap	n.s.	n.s.	**d ≥ 1:30**	**d ≥ 1:30**	d 1:10	1,07	1,03	1,05	146,54	1,40	1,39	1,39	128,54	0,32	0,33	0,32	24,97
**MR 245 /15 SR**	Tagant	Letvetar	n.s.	cap	n.s.	n.s.	**d ≥ 1:30**	**d ≥ 1:30**	d 1:10	0,25	0,26	0,26	35,49	0,99	0,99	0,99	91,33	1,16	1,19	1,18	92,68
**MR 247 /15 SR**	Tagant	Letvetar	n.s.	cap	n.s.	n.s.	d < 1:10	d < 1:10	d < 1:10	0,15	0,14	0,14	20,10	0,24	0,24	0,24	22,18	0,25	0,24	0,24	19,26
**MR 249 /15 SR**	Tagant	Letvetar	n.s.	cap	n.s.	n.s.	**d ≥ 1:30**	**d ≥ 1:30**	d < 1:10	0,23	0,20	0,22	30,06	0,63	0,61	0,62	56,53	0,27	0,25	0,26	20,58
**MR 250 /15 SR**	Tagant	Letvetar	n.s.	cap	n.s.	n.s.	d < 1:10	d < 1:10	d 1:10	0,16	0,14	0,15	20,45	0,21	0,21	0,21	19,26	1,67	1,61	1,64	128,91

**MR 251 /15 SR**	Tagant	Letvetar	n.s.	cap	n.s.	n.s.	**d ≥ 1:30**	**d ≥ 1:60**	d < 1:10	0,54	0,46	0,50	69,21	0,44	0,43	0,43	39,48	0,72	0,67	0,70	54,79
**MR 252 /15 SR**	Tagant	Letvetar	n.s.	cap	n.s.	n.s.	**d ≥ 1:30**	**d ≥ 1:60**	d 1:10	1,20	1,12	1,16	161,59	1,33	1,33	1,33	121,42	1,02	1,05	1,04	81,58
**MR 254 /15 SR**	Tagant	Letvetar	n.s.	ov	n.s.	n.s.	**d ≥ 1:30**	**d ≥ 1:60**	d < 1:10	0,18	0,17	0,18	24,88	0,54	0,51	0,53	48,08	0,50	0,48	0,49	38,50
**MR 255 /15 SR**	Tagant	Letvetar	n.s.	ov	n.s.	n.s.	d < 1:10	d < 1:10	d < 1:10	0,13	0,12	0,13	17,78	0,17	0,22	0,19	17,78	0,68	0,79	0,74	57,89
**MR 256 /15 SR**	Tagant	Letvetar	n.s.	cap	n.s.	n.s.	d < 1:10	d < 1:10	d < 1:10	0,10	0,09	0,09	13,09	0,13	0,12	0,13	11,58	0,27	0,28	0,28	21,75
**MR 260 /15 SR**	Tagant	Letvetar	n.s.	ov	n.s.	n.s.	**d ≥ 1:30**	**d ≥ 1:30**	d < 1:10	1,28	1,27	1,28	177,58	1,04	1,00	1,02	93,12	0,27	0,27	0,27	21,11
**MR 262 /15 SR**	Tagant	Letvetar	n.s.	cap	n.s.	n.s.	**d ≥ 1:30**	**d ≥ 1:30**	d < 1:10	0,26	0,26	0,26	36,21	0,29	0,29	0,29	26,21	0,59	0,60	0,60	46,96
**MR 263 /15 SR**	Tagant	Letvetar	n.s.	cap	n.s.	n.s.	**d ≥ 1:30**	**d ≥ 1:30**	d < 1:10	1,44	1,40	1,42	197,61	1,90	1,94	1,92	175,24	0,71	0,70	0,71	55,74
**MR 264 /15 SR**	Tagant	Letvetar	n.s.	cap	n.s.	n.s.	d < 1:10	d < 1:10	d < 1:10	0,10	0,09	0,10	13,30	0,27	0,27	0,27	24,71	0,38	0,38	0,38	30,02
**MR 265 /15 SR**	Tagant	Letvetar	n.s.	cap	n.s.	n.s.	**d ≥ 1:30**	**d ≥ 1:30**	d 1:10	0,75	0,79	0,77	107,20	1,00	0,96	0,98	89,20	0,22	0,21	0,22	16,95
**MR 266 /15 SR**	Tagant	Letvetar	n.s.	cap	n.s.	n.s.	**d ≥ 1:30**	**d ≥ 1:30**	d 1:10	1,66	1,70	1,68	233,74	1,91	1,96	1,94	176,68	0,33	0,32	0,33	25,85
**MR 267 /15 SR**	Tagant	Letvetar	n.s.	cap	n.s.	n.s.	d < 1:10	d < 1:10	d < 1:10	0,09	0,09	0,09	12,82	0,15	0,16	0,15	14,13	0,35	0,35	0,35	27,78
**MR 268 /15 SR**	Tagant	Letvetar	n.s.	cap	n.s.	n.s.	d < 1:10	d < 1:10	d < 1:10	0,12	0,11	0,12	16,08	0,15	0,15	0,15	13,75	0,36	0,35	0,36	28,09
**MR 269 /15 SR**	Tagant	Letvetar	n.s.	cap	n.s.	n.s.	**d ≥ 1:30**	**d ≥ 1:30**	d < 1:10	0,74	0,71	0,72	100,72	1,02	1,03	1,02	93,50	0,38	0,37	0,38	29,64
**MR 270 /15 SR**	Tagant	Letvetar	n.s.	cap	n.s.	n.s.	d < 1:10	d < 1:10	d < 1:10	0,11	0,11	0,11	14,97	0,17	0,17	0,17	15,57	0,37	0,36	0,37	28,87
**MR 271 /15 SR**	Tagant	Letvetar	n.s.	cap	n.s.	n.s.	**d ≥ 1:30**	**d ≥ 1:30**	d < 1:10	1,34	1,33	1,34	185,81	1,39	1,40	1,39	127,21	0,43	0,44	0,43	33,91
**MR 273 /15 SR**	Tagant	Gnimlane/ Deroom	n.s.	cap	f	3	d < 1:10	d < 1:10	d < 1:10	0,13	0,12	0,12	17,31	0,14	0,14	0,14	12,56	0,25	0,22	0,24	18,55
**MR 274 /15 SR**	Tagant	Gnimlane/ Deroom	n.s.	cap	f	2	d < 1:10	d < 1:10	d < 1:10	0,11	0,11	0,11	15,36	0,13	0,13	0,13	11,58	0,16	0,16	0,16	12,75
**MR 275 /15 SR**	Tagant	Gnimlane/ Deroom	n.s.	cap	f	1	d < 1:10	d < 1:10	d < 1:10	0,12	0,12	0,12	16,48	0,17	0,17	0,17	15,57	0,18	0,18	0,18	14,34
**MR 276 /15 SR**	Tagant	Gnimlane/ Deroom	n.s.	cap	f	3	d < 1:10	d < 1:10	d < 1:10	0,09	0,09	0,09	12,75	0,10	0,10	0,10	9,10	0,22	0,23	0,23	17,72
**MR 277 /15 SR**	Tagant	Gnimlane/ Deroom	n.s.	cap	f	3	d < 1:10	d < 1:10	d < 1:10	0,14	0,15	0,15	20,36	0,18	0,18	0,18	16,69	0,45	0,46	0,45	35,71
**MR 278 /15 SR**	Tagant	Gnimlane/ Deroom	n.s.	cap	f	2	d < 1:10	d < 1:10	d < 1:10	0,21	0,21	0,21	29,02	0,22	0,22	0,22	20,10	0,52	0,55	0,53	41,93
**MR 279 /15 SR**	Tagant	Gnimlane/ Deroom	n.s.	cap	f	2	d < 1:10	d < 1:10	d < 1:10	0,10	0,11	0,10	14,58	0,11	0,11	0,11	10,44	0,28	0,29	0,28	22,29
**MR 280 /15 SR**	Tagant	Gnimlane/ Deroom	n.s.	cap	f	3	d < 1:10	d < 1:10	d < 1:10	0,20	0,20	0,20	25,96	0,15	0,17	0,16	14,64	0,26	0,28	0,27	21,26
**MR 281 /15 SR**	Tagant	Gnimlane/ Deroom	n.s.	cap	f	1	**d ≥ 1:30**	**d ≥ 1:60**	d 1:20	1,44	1,44	1,44	187,53	1,47	1,45	1,46	133,09	0,32	0,34	0,33	25,89
**MR 283 /15 SR**	Tagant	Gnimlane/ Deroom	n.s.	cap	f	2	d < 1:10	d < 1:10	d < 1:10	0,10	0,09	0,09	12,14	0,17	0,15	0,16	14,59	0,36	0,38	0,37	29,29
**MR 284 /15 SR**	Tagant	Gnimlane/ Deroom	n.s.	cap	f	3	d < 1:10	d < 1:10	d < 1:10	0,16	0,13	0,14	18,65	0,17	0,17	0,17	15,50	0,58	0,61	0,59	46,78
**MR 285 /15 SR**	Tagant	Gnimlane/ Deroom	n.s.	cap	f	3	d < 1:10	d < 1:10	d < 1:10	0,45	0,34	0,39	51,14	0,42	0,43	0,43	39,11	0,59	0,59	0,59	46,50
**MR 286 /15 SR**	Tagant	Gnimlane/ Deroom	n.s.	cap	f	1	d < 1:10	d < 1:10	d < 1:10	0,15	0,14	0,14	18,64	0,19	0,18	0,19	16,95	0,40	0,45	0,42	33,33
**MR 287 /15 SR**	Tagant	Gnimlane/ Deroom	n.s.	cap	f	2	d < 1:10	d < 1:10	d < 1:10	0,10	0,09	0,10	12,60	0,13	0,14	0,13	12,32	0,34	0,35	0,34	27,09
**MR 288 /15 SR**	Tagant	Gnimlane/ Deroom	n.s.	cap	f	3	d < 1:10	d < 1:10	d < 1:10	0,08	0,08	0,08	9,93	0,08	0,08	0,08	7,66	0,13	0,13	0,13	10,36
**MR 289 /15 SR**	Tagant	Gnimlane/ Deroom	n.s.	cap	f	3	d < 1:10	d < 1:10	d < 1:10	0,08	0,08	0,08	10,58	0,11	0,11	0,11	10,13	0,22	0,24	0,23	18,30
**MR 290 /15 SR**	Tagant	Gnimlane/ Deroom	n.s.	cap	f	2	d < 1:10	d < 1:10	d < 1:10	0,12	0,12	0,12	15,70	0,16	0,15	0,15	14,03	0,40	0,42	0,41	32,17
**MR 291 /15 SR**	Tagant	Gnimlane/ Deroom	n.s.	cap	f	2	d < 1:10	d < 1:10	d < 1:10	0,10	0,11	0,11	13,76	0,28	0,29	0,28	26,09	0,22	0,23	0,23	17,94
**MR 292 /15 SR**	Tagant	Gnimlane/ Deroom	n.s.	cap	f	3	d < 1:10	d < 1:10	d < 1:10	0,19	0,14	0,17	21,83	0,17	0,16	0,16	14,93	0,64	0,70	0,67	52,81
**MR 293 /15 SR**	Tagant	Gnimlane/ Deroom	n.s.	cap	f	2	d < 1:10	d < 1:10	d < 1:10	0,09	0,09	0,09	11,93	0,16	0,16	0,16	14,62	0,34	0,37	0,36	28,29
**MR 294 /15 SR**	Tagant	Gnimlane/ Deroom	n.s.	cap	m	1	d < 1:10	d < 1:10	d < 1:10	0,13	0,13	0,13	17,33	0,24	0,24	0,24	22,11	0,26	0,27	0,27	21,44
**MR 295 /15 SR**	Tagant	Gnimlane/ Deroom	n.s.	cap	f	2	d < 1:10	d < 1:10	d < 1:10	0,21	0,19	0,20	25,87	0,25	0,26	0,25	23,51	0,27	0,27	0,27	21,71
**MR 296 /15 SR**	Tagant	Gnimlane/ Deroom	n.s.	cap	f	2	d < 1:10	d < 1:10	d < 1:10	0,10	0,10	0,10	13,03	0,13	0,13	0,13	11,97	0,20	0,20	0,20	16,28
**MR 297 /15 SR**	Tagant	Gnimlane/ Deroom	n.s.	cap	f	1	d < 1:10	d < 1:10	d < 1:10	0,14	0,13	0,14	18,10	0,22	0,21	0,22	19,99	0,18	0,18	0,18	14,27
**MR 298 /15 SR**	Tagant	Gnimlane/ Deroom	n.s.	cap	f	2	d < 1:10	d < 1:10	d < 1:10	0,09	0,09	0,09	11,62	0,13	0,20	0,17	15,47	0,41	0,42	0,41	33,33
**MR 299 /15 SR**	Tagant	Gnimlane/ Deroom	n.s.	cap	f	3	d < 1:10	d < 1:10	d < 1:10	0,11	0,10	0,10	13,65	0,17	0,17	0,17	14,36	0,24	0,24	0,24	19,73
**MR 300 /15 SR**	Tagant	Gnimlane/ Deroom	n.s.	cap	f	3	d < 1:10	d < 1:10	d < 1:10	0,10	0,10	0,10	12,85	0,16	0,15	0,15	12,70	0,28	0,22	0,25	20,30
**MR 301 /15 SR**	Tagant	Gnimlane/ Deroom	n.s.	cap	f	2	d < 1:10	d < 1:10	d < 1:10	0,11	0,11	0,11	14,74	0,15	0,14	0,15	12,14	0,35	0,35	0,35	28,45
**MR 302 /15 SR**	Tagant	Gnimlane/ Deroom	n.s.	cap	f	2	d < 1:10	d < 1:10	d < 1:10	0,08	0,08	0,08	10,84	0,15	0,14	0,14	12,06	0,74	0,82	0,78	62,92
**MR 303 /15 SR**	Tagant	Gnimlane/ Deroom	n.s.	cap	f	2	d < 1:10	d < 1:10	d < 1:10	0,10	0,10	0,10	13,37	0,14	0,14	0,14	11,29	0,26	0,25	0,26	20,71
**MR 304 /15 SR**	Assaba	Bougamara	n.s.	ov	f	3	**d 1:20**	**d 1:40**	d < 1:10	0,13	0,13	0,13	16,51	0,12	0,12	0,12	10,20	0,26	0,26	0,26	20,87
**MR 305 /15 SR**	Assaba	Bougamara	n.s.	ov	f	4	**d ≥ 1:30**	**d ≥ 1:60**	d 1:10	0,38	0,36	0,37	48,48	0,48	0,48	0,48	39,95	0,30	0,30	0,30	24,39
**MR 306 /15 SR**	Assaba	Bougamara	n.s.	ov	f	5	d < 1:10	d < 1:10	d < 1:10	0,10	0,09	0,10	12,43	0,49	0,51	0,50	41,67	0,48	0,45	0,47	37,68
**MR 308 /15 SR**	Assaba	Bougamara	n.s.	ov	f	2	**d ≥ 1:30**	**d ≥ 1:30**	d < 1:10	0,19	0,18	0,18	24,07	0,36	0,32	0,34	28,38	0,50	0,51	0,51	40,91
**MR 309 /15 SR**	Assaba	Bougamara	n.s.	ov	m	4	**d ≥ 1:30**	**d ≥ 1:30**	d 1:10	0,54	0,46	0,50	65,57	0,53	0,52	0,53	43,95	0,61	0,58	0,59	48,02
**MR 310 /15 SR**	Assaba	Bougamara	n.s.	ov	f	1	**d ≥ 1:30**	**d ≥ 1:30**	d 1:10	0,33	0,30	0,32	41,45	0,36	0,34	0,35	29,41	0,49	0,42	0,46	36,91
**MR 311 /15 SR**	Assaba	Bougamara	n.s.	ov	f	4	**d ≥ 1:30**	**d ≥ 1:30**	d 1:10	0,14	0,14	0,14	17,95	0,17	0,17	0,17	14,07	0,44	0,41	0,42	34,10
**MR 312 /15 SR**	Assaba	Bougamara	n.s.	ov	f	3	d < 1:10	d < 1:10	d < 1:10	0,09	0,09	0,09	11,39	0,10	0,10	0,10	8,20	0,19	0,18	0,18	14,71
**MR 314 /15 SR**	Assaba	Bougamara	n.s.	ov	f	4	d < 1:10	d < 1:10	d < 1:10	0,14	0,13	0,14	17,80	0,16	0,16	0,16	13,73	0,26	0,26	0,26	21,08
**MR 315 /15 SR**	Assaba	Bougamara	n.s.	ov	f	3	**d ≥ 1:30**	**d ≥ 1:30**	d < 1:10	0,11	0,10	0,10	13,35	0,16	0,16	0,16	13,74	0,33	0,33	0,33	26,75
**MR 316 /15 SR**	Assaba	Bougamara	n.s.	ov	f	3	d < 1:10	d < 1:10	d < 1:10	0,12	0,12	0,12	15,30	0,16	0,15	0,16	13,27	0,27	0,26	0,27	21,69
**MR 317 /15 SR**	Assaba	Bougamara	n.s.	ov	f	2	**d ≥ 1:30**	**d ≥ 1:30**	d < 1:10	0,27	0,24	0,26	33,36	0,28	0,28	0,28	23,28	0,46	0,43	0,45	35,96
**MR 319 /15 SR**	Assaba	Bougamara	n.s.	ov	f	4	**d ≥ 1:30**	**d ≥ 1:30**	d 1:10	0,49	0,42	0,46	59,34	0,55	0,53	0,54	44,84	0,42	0,41	0,41	33,32
**MR 320 /15 SR**	Assaba	Bougamara	n.s.	ov	f	3	**d ≥ 1:30**	**d ≥ 1:30**	d 1:10	0,25	0,23	0,24	31,35	0,26	0,25	0,26	21,51	0,37	0,36	0,36	29,42
**MR 321 /15 SR**	Assaba	Bougamara	n.s.	ov	f	1	**d ≥ 1:30**	**d ≥ 1:30**	d < 1:10	0,26	0,24	0,25	32,66	0,36	0,37	0,37	30,63	0,20	0,20	0,20	16,03
**MR 322 /15 SR**	Assaba	Bougamara	n.s.	ov	f	4	d < 1:10	d < 1:10	d < 1:10	0,08	0,08	0,08	10,53	0,11	0,10	0,10	8,74	0,50	0,52	0,51	41,51

MR 323 /15 SR	Assaba	Bougamara	n.s.	ov	f	5	d ≥ 1:30	d 1:40	d < 1:10	0,16	0,14	0,15	19,06	0,14	0,13	0,14	11,34	0,25	0,25	0,25	20,21
MR 324 /15 SR	Assaba	Bougamara	n.s.	ov	f	3	d ≥ 1:30	d 1:40	d 1:10	0,50	0,42	0,46	59,84	0,65	0,63	0,64	53,17	1,96	2,03	1,99	161,05
MR 325 /15 SR	Assaba	Bougamara	n.s.	ov	m	4	d < 1:10	d < 1:10	d < 1:10	0,11	0,11	0,11	14,59	0,22	0,23	0,22	18,69	0,50	0,51	0,50	40,73
MR 326 /15 SR	Assaba	Bougamara	n.s.	ov	f	5	d ≥ 1:30	d ≥ 1:60	d 1:10	0,37	0,36	0,37	47,02	0,31	0,29	0,30	25,14	0,34	0,33	0,34	27,11
MR 327 /15 SR	Assaba	Bougamara	n.s.	ov	m	5	d ≥ 1:30	d 1:20	d < 1:10	0,36	0,37	0,37	47,37	0,34	0,33	0,33	27,80	0,20	0,21	0,21	16,56
MR 328 /15 SR	Assaba	Bougamara	n.s.	ov	f	3	d 1:10	d 1:30	d < 1:10	0,10	0,10	0,10	12,79	0,14	0,14	0,14	11,46	0,43	0,42	0,42	34,00
MR 329 /15 SR	Assaba	Bougamara	n.s.	ov	f	3	d ≥ 1:30	d ≥ 1:60	d < 1:10	0,25	0,24	0,24	31,48	0,40	0,39	0,40	33,13	0,40	0,44	0,42	34,09
MR 332 /15 SR	Assaba	Bougamara	n.s.	ov	f	3	d ≥ 1:30	d ≥ 1:60	d < 1:10	0,67	0,61	0,64	82,36	1,66	1,60	1,63	135,98	1,66	1,68	1,67	134,91
MR 335 /15 SR	Assaba	Bougamara	n.s.	ov	f	3	d ≥ 1:30	d ≥ 1:30	d < 1:10	0,26	0,25	0,26	32,94	0,45	0,45	0,45	37,50	0,27	0,27	0,27	21,71
MR 336 /15 SR	Assaba	Tamourett Voulane	n.s.	cap	f	4	d 1:120	d ≥ 1:30	d 1:10	0,89	0,75	0,82	104,92	0,93	0,88	0,90	75,36	0,93	1,09	1,01	81,54
MR 337 /15 SR	Assaba	Tamourett Voulane	n.s.	cap	f	4	d ≥ 1:30	d ≥ 1:30	d < 1:10	1,13	1,14	1,13	145,82	1,13	1,15	1,14	95,45	0,70	0,75	0,72	58,46
MR 338 /15 SR	Assaba	Tamourett Voulane	n.s.	cap	f	3	d 1:20	d < 1:10	d < 1:10	0,12	0,12	0,12	15,71	0,20	0,21	0,20	17,11	0,30	0,29	0,29	23,48
MR 339 /15 SR	Assaba	Tamourett Voulane	n.s.	cap	f	5	d ≥ 1:30	d ≥ 1:30	d 1:10	0,93	0,87	0,90	116,03	1,21	1,26	1,24	103,16	1,45	1,65	1,55	125,00
MR 341 /15 SR	Assaba	Tamourett Voulane	n.s.	cap	f	1	d < 1:10	d < 1:10	d < 1:10	0,09	0,08	0,09	6,36	0,14	0,16	0,15	10,08	0,53	0,53	0,53	37,12
MR 342 /15 SR	Assaba	Tamourett Voulane	n.s.	cap	f	4	d ≥ 1:30	d ≥ 1:30	d < 1:10	0,68	0,69	0,68	87,87	1,15	1,16	1,16	96,65	1,02	0,99	1,00	70,36
MR 343 /15 SR	Assaba	Tamourett Voulane	n.s.	cap	f	1	d < 1:10	d < 1:10	d < 1:10	0,09	0,09	0,09	11,70	0,13	0,13	0,13	10,90	0,28	0,29	0,28	19,78
MR 344 /15 SR	Assaba	Tamourett Voulane	n.s.	cap	f	2	d < 1:10	d < 1:10	d < 1:10	0,10	0,10	0,10	12,81	0,13	0,13	0,13	11,20	0,57	0,57	0,57	39,94
MR 345 /15 SR	Assaba	Tamourett Voulane	n.s.	cap	f	1	d < 1:10	d < 1:10	d < 1:10	0,10	0,10	0,10	13,05	0,12	0,12	0,12	10,21	0,51	0,51	0,51	35,44
MR 346 /15 SR	Assaba	Tamourett Voulane	n.s.	cap	f	1	d < 1:10	d < 1:10	d < 1:10	0,25	0,20	0,23	16,11	0,42	0,37	0,40	26,82	0,81	0,77	0,79	55,42
MR 347 /15 SR	Assaba	Tamourett Voulane	n.s.	cap	f	1	d < 1:10	d < 1:10	d < 1:10	0,10	0,10	0,10	10,23	0,28	0,30	0,29	19,72	0,32	0,32	0,32	22,49
MR 348 /15 SR	Assaba	Tamourett Voulane	n.s.	ov	f	1	d < 1:10	d < 1:10	d < 1:10	0,08	0,08	0,08	10,29	0,11	0,14	0,13	10,64	0,24	0,25	0,25	17,46
MR 349 /15 SR	Assaba	Tamourett Voulane	n.s.	cap	f	1	d < 1:10	d < 1:10	d < 1:10	0,10	0,08	0,09	6,54	0,12	0,15	0,14	9,16	0,17	0,17	0,17	11,77
MR 350 /15 SR	Assaba	Tamourett Voulane	n.s.	cap	f	5	d ≥ 1:30	d ≥ 1:30	d < 1:10	1,11	1,04	1,07	137,86	1,66	1,57	1,61	132,71	0,66	0,67	0,66	46,42
MR 351 /15 SR	Assaba	Tamourett Voulane	n.s.	cap	f	4	d < 1:10	d < 1:10	d < 1:10	0,13	0,13	0,13	16,67	0,27	0,26	0,26	21,47	0,48	0,47	0,48	33,41
MR 352 /15 SR	Assaba	Tamourett Voulane	n.s.	cap	f	6	d 1:160	d 1:80	d 1:20	1,63	1,02	1,32	94,73	1,38	1,23	1,31	88,29	0,83	0,80	0,81	57,01
MR 353 /15 SR	Assaba	Tamourett Voulane	n.s.	cap	f	3	d 1:80	d 1:60	d < 1:10	0,90	0,35	0,62	44,66	1,25	1,38	1,31	88,87	0,97	0,96	0,97	67,59
MR 354 /15 SR	Assaba	Tamourett Voulane	n.s.	cap	f	1	d < 1:10	d < 1:10	d < 1:10	0,18	0,12	0,15	19,64	0,18	0,17	0,17	14,28	0,46	0,46	0,46	32,47
MR 355 /15 SR	Assaba	Tamourett Voulane	n.s.	cap	m	1	d 1:10	d < 1:10	d < 1:10	0,09	0,09	0,09	11,20	0,09	0,10	0,10	7,82	0,19	0,19	0,19	13,40
MR 356 /15 SR	Assaba	Tamourett Voulane	n.s.	cap	m	1	d < 1:10	d < 1:10	d < 1:10	0,08	0,08	0,08	10,27	0,10	0,10	0,10	8,40	0,18	0,18	0,18	12,63
MR 357 /15 SR	Assaba	Tamourett Voulane	n.s.	cap	f	4	d ≥ 1:30	d 1:20	d 1:10	0,23	0,23	0,23	29,12	0,40	0,40	0,40	32,84	0,21	0,22	0,21	16,78
MR 358 /15 SR	Assaba	Tamourett Voulane	n.s.	cap	f	3	d ≥ 1:30	d 1:40	d < 1:10	0,40	0,38	0,39	50,03	0,65	0,63	0,64	52,68	0,67	0,71	0,69	54,19
MR 359 /15 SR	Assaba	Tamourett Voulane	n.s.	cap	f	4	d ≥ 1:30	d ≥ 1:60	d 1:10	0,35	0,35	0,35	44,94	0,72	0,70	0,71	58,58	0,46	0,46	0,46	32,26
MR 360 /15 SR	Assaba	Tamourett Voulane	n.s.	cap	f	4	d ≥ 1:30	d ≥ 1:60	d 1:10	0,60	0,54	0,57	72,91	0,92	0,89	0,91	74,75	0,44	0,43	0,43	30,30
MR 361 /15 SR	Assaba	Tamourett Voulane	n.s.	cap	f	4	d ≥ 1:30	d ≥ 1:60	d 1:30	0,27	0,25	0,26	33,89	0,43	0,42	0,43	35,21	0,77	0,79	0,78	54,57
MR 362 /15 SR	Assaba	Tamourett Voulane	n.s.	ov	f	1	d < 1:10	d < 1:10	d < 1:10	0,55	0,53	0,54	69,47	0,85	0,86	0,86	70,50	0,60	0,60	0,60	42,06
MR 363 /15 SR	Assaba	Tamourett Voulane	n.s.	cap	f	4	d < 1:10	d 1:40	d < 1:10	0,55	0,54	0,54	69,83	0,99	0,97	0,98	80,52	0,76	0,75	0,76	52,92
MR 364 /15 SR	Assaba	Tamourett Voulane	n.s.	cap	f	1	d < 1:10	d < 1:10	d < 1:10	0,10	0,10	0,10	12,30	0,16	0,15	0,15	12,69	0,40	0,40	0,40	27,87
MR 365 /15 SR	Assaba	Tamourett Voulane	n.s.	cap	f	1	d ≥ 1:30	d < 1:10	d < 1:10	0,08	0,08	0,08	10,13	0,16	0,16	0,16	13,31	0,35	0,35	0,35	24,53
MR 366 /15 SR	Trarza	N'Diourbel	n.s.	ov	f	2	d 1:10	d < 1:10	d < 1:10	0,10	0,10	0,10	12,81	0,11	0,11	0,11	8,82	0,27	0,28	0,28	19,30
MR 367 /15 SR	Trarza	N'Diourbel	n.s.	ov	m	1	d < 1:10	d < 1:10	d < 1:10	0,09	0,09	0,09	11,04	0,12	0,12	0,12	9,64	0,19	0,20	0,19	13,47
MR 368 /15 SR	Trarza	N'Diourbel	n.s.	ov	f	1	d 1:10	d 1:15	d < 1:10	0,13	0,13	0,13	16,32	0,25	0,23	0,24	19,80	0,89	0,91	0,90	62,78
MR 369 /15 SR	Trarza	N'Diourbel	n.s.	cap	m	2	d < 1:10	d < 1:10	d < 1:10	0,12	0,12	0,12	15,52	0,14	0,13	0,13	10,93	0,36	0,39	0,38	26,38
MR 370 /15 SR	Trarza	N'Diourbel	n.s.	cap	f	3	d ≥ 1:30	d < 1:10	d < 1:10	0,26	0,28	0,27	34,71	0,20	0,20	0,20	16,38	0,75	0,77	0,76	53,27
MR 371 /15 SR	Trarza	N'Diourbel	n.s.	ov	f	1	d ≥ 1:30	d ≥ 1:30	d < 1:10	0,22	0,22	0,22	28,77	0,31	0,31	0,31	25,39	0,66	0,67	0,67	46,61
MR 372 /15 SR	Trarza	N'Diourbel	n.s.	ov	m	1	d < 1:10	d ≥ 1:30	d < 1:10	0,08	0,08	0,08	10,72	0,09	0,09	0,09	7,38	0,81	0,83	0,82	57,43
MR 373 /15 SR	Trarza	N'Diourbel	n.s.	cap	f	3	d ≥ 1:30	d ≥ 1:30	d < 1:10	0,32	0,30	0,31	40,07	0,49	0,49	0,49	40,24	0,73	0,78	0,75	52,76
MR 374 /15 SR	Trarza	N'Diourbel	n.s.	ov	f	2	d < 1:10	d < 1:10	d < 1:10	0,16	0,16	0,16	20,44	0,21	0,21	0,21	16,93	0,52	0,50	0,51	35,75
MR 375 /15 SR	Trarza	N'Diourbel	n.s.	ov	f	2	d ≥ 1:30	d ≥ 1:30	d < 1:10	0,50	0,46	0,48	61,62	0,81	0,79	0,80	65,78	0,33	0,32	0,32	22,57
MR 376 /15 SR	Trarza	N'Diourbel	n.s.	ov	m	1	d < 1:10	d < 1:10	d < 1:10	0,12	0,13	0,12	16,02	0,17	0,15	0,16	13,04	0,33	0,32	0,33	22,87
MR 377 /15 SR	Trarza	N'Diourbel	n.s.	ov	f	2	d < 1:10	d < 1:10	d < 1:10	0,16	0,16	0,16	20,09	0,21	0,21	0,21	17,26	0,42	0,40	0,41	28,76
MR 378 /15 SR	Trarza	N'Diourbel	n.s.	cap	f	3	d 1:160	d 1:320	d 1:10	0,49	0,40	0,45	32,01	0,44	0,39	0,42	28,20	0,58	0,59	0,59	40,98
MR 379 /15 SR	Trarza	N'Diourbel	n.s.	cap	f	3	d 1:60	d 1:60	evtl. d 1:10	1,91	1,57	1,74	124,24	1,36	1,71	1,53	103,69	0,35	0,34	0,34	24,16
MR 380 /15 SR	Trarza	N'Diourbel	n.s.	ov	f	3	d < 1:10	d < 1:10	d < 1:10	0,08	0,08	0,08	11,09	0,14	0,10	0,12	9,71	0,26	0,29	0,27	19,08
MR 381 /15 SR	Trarza	N'Diourbel	n.s.	cap	f	3	d < 1:10	d < 1:10	d < 1:10	0,26	0,23	0,24	33,78	0,55	0,55	0,55	45,18	0,96	1,03	1,00	75,43
MR 383 /15 SR	Trarza	N'Diourbel	n.s.	cap	f	4	d ≥ 1:30	d 1:40	d < 1:10	0,46	0,44	0,45	62,48	0,79	0,77	0,78	63,89	0,59	0,58	0,58	43,98
MR 384 /15 SR	Trarza	N'Diourbel	n.s.	ov	m	1	d < 1:10	d < 1:10	d < 1:10	0,09	0,07	0,08	11,25	0,08	0,09	0,08	6,97	0,42	0,42	0,42	31,74
MR 385 /15 SR	Trarza	N'Diourbel	n.s.	ov	f	2	d ≥ 1:30	d 1:40	d 1:20	0,41	0,40	0,40	55,66	0,61	0,62	0,61	50,29	0,34	0,36	0,35	26,26
MR 386 /15 SR	Trarza	N'Diourbel	n.s.	cap	m	1	d < 1:10	d < 1:10	d < 1:10	0,12	0,12	0,12	16,94	0,22	0,25	0,23	19,16	0,38	0,39	0,38	28,87
MR 387 /15 SR	Trarza	N'Diourbel	n.s.	ov	f	2	d < 1:10	d < 1:10	d < 1:10	0,09	0,08	0,08	11,30	0,10	0,11	0,10	8,57	0,45	0,48	0,46	35,13
MR 388 /15 SR	Trarza	N'Diourbel	n.s.	ov	m	2	d < 1:10	d < 1:10	d < 1:10	0,08	0,08	0,08	10,74	0,09	0,10	0,09	7,70	0,17	0,16	0,16	12,36
MR 389 /15 SR	Trarza	N'Diourbel	n.s.	ov	f	3	d < 1:10	d < 1:10	d < 1:10	0,08	0,08	0,08	10,89	0,09	0,09	0,09	7,37	0,28	0,27	0,28	20,91
MR 390 /15 SR	Trarza	N'Diourbel	n.s.	ov	f	1	d < 1:10	d < 1:10	d < 1:10	0,08	0,08	0,08	11,09	0,09	0,10	0,09	7,77	0,37	0,38	0,38	28,55

**MR 392 /15 SR**	Trarza	N'Diourbel	n.s.	cap	m	2	**d ≥ 1:30**	**d 1:20**	d < 1:10	0,53	0,53	0,53	73,40	0,63	0,62	0,63	51,65	1,48	1,49	1,49	112,47
**MR 394 /15 SR**	Trarza	N'Diourbel	n.s.	ov	m	2	**d 1:1280**	**d 1:960**	d < 1:10	1,47	1,00	1,24	88,46	2,41	2,00	2,20	149,18	0,86	0,89	0,88	66,53
**MR 395 /15 SR**	Trarza	N'Diourbel	n.s.	cap	m	1	d < 1:10	d < 1:10	d < 1:10	0,09	0,09	0,09	12,09	0,11	0,12	0,12	9,61	0,16	0,16	0,16	12,20
**MR 396 /15 SR**	Trarza	N'Diourbel	n.s.	ov	f	1	**d ≥ 1:30**	**d ≥ 1:30**	d 1:10	0,13	0,14	0,13	18,48	0,31	0,36	0,34	27,74	1,41	1,42	1,41	106,81
**MR 397 /15 SR**	Guidimaka	Mamayel	n.s.	ov	f	3	**d ≥ 1:30**	**d ≥ 1:30**	d < 1:10	0,20	0,20	0,20	27,68	0,41	0,38	0,39	32,07	0,87	0,88	0,87	66,08
**MR 398 /15 SR**	Guidimaka	Mamayel	n.s.	ov	f	2	**d ≥ 1:30**	**d ≥ 1:30**	d < 1:10	0,39	0,35	0,37	51,37	0,72	0,69	0,70	57,23	0,88	0,87	0,87	66,22
**MR 399 /15 SR**	Guidimaka	Mamayel	n.s.	ov	f	3	**d ≥ 1:30**	**d ≥ 1:30**	d < 1:10	0,66	0,67	0,67	92,54	1,02	1,04	1,03	83,63	1,75	1,71	1,73	130,90
**MR 400 /15 SR**	Guidimaka	Mamayel	n.s.	ov	f	2	**d ≥ 1:30**	**d ≥ 1:30**	d 1:10	0,68	0,71	0,69	96,26	1,46	1,44	1,45	117,66	0,31	0,29	0,30	22,66
**MR 401 /15 SR**	Guidimaka	Mamayel	n.s.	ov	f	2	**d 1:40**	**d 1:30**	d < 1:10	0,36	0,20	0,28	20,03	0,78	0,56	0,67	45,18	0,42	0,42	0,42	31,84
**MR 402 /15 SR**	Guidimaka	Mamayel	n.s.	ov	m	3	**d 1:240**	**d 1:320**	d < 1:10	2,16	1,70	1,93	137,98	1,62	1,46	1,54	104,14	0,59	0,59	0,59	44,64
**MR 403 /15 SR**	Guidimaka	Mamayel	n.s.	ov	f	2	d < 1:10	d < 1:10	d < 1:10	0,10	0,10	0,10	13,72	0,16	0,16	0,16	13,38	0,50	0,49	0,49	37,19
**MR 404 /15 SR**	Guidimaka	Mamayel	n.s.	ov	f	3	**d ≥ 1:30**	**d ≥ 1:30**	d 1:10	0,80	0,77	0,78	108,51	1,10	1,13	1,12	90,80	0,32	0,33	0,32	24,53
**MR 405 /15 SR**	Guidimaka	Mamayel	n.s.	ov	f	4	**d ≥ 1:120**	**d ≥ 1:30**	d < 1:10	1,01	0,96	0,98	136,17	1,40	1,39	1,39	113,35	1,07	1,11	1,09	82,33
**MR 406 /15 SR**	Guidimaka	Mamayel	n.s.	ov	m	2	**d ≥ 1:120**	**d ≥ 1:30**	d 1:10	1,85	1,78	1,81	251,44	2,81	2,86	2,83	230,48	2,09	2,11	2,10	158,70
**MR 407 /15 SR**	Guidimaka	Mamayel	n.s.	ov	f	3	**d 1:60**	**d 1:60**	d < 1:10	0,54	0,55	0,55	39,31	0,74	0,80	0,77	52,19	0,51	0,50	0,50	38,20
**MR 408 /15 SR**	Guidimaka	Mamayel	n.s.	ov	f	3	d < 1:10	d < 1:10	d < 1:10	0,10	0,15	0,13	9,00	0,13	0,36	0,25	16,72	0,31	0,32	0,32	23,87
**MR 409 /15 SR**	Guidimaka	Mamayel	n.s.	ov	f	4	d < 1:10	d < 1:10	d < 1:10	0,10	0,10	0,10	7,22	0,13	0,40	0,27	18,09	0,70	0,73	0,71	54,13
**MR 410 /15 SR**	Guidimaka	Mamayel	n.s.	ov	f	4	d < 1:10	d < 1:10	d < 1:10	0,10	0,09	0,10	6,98	0,13	0,15	0,14	9,50	0,27	0,28	0,27	20,80
**MR 413 /15 SR**	Guidimaka	Mamayel	n.s.	cap	f	2	**d 1:80**	**d ≥ 1:30**	d < 1:10	0,30	0,31	0,30	42,24	0,53	0,51	0,52	41,96	0,51	0,52	0,51	38,88
**MR 414 /15 SR**	Guidimaka	Mamayel	n.s.	cap	f	2	d < 1:10	d < 1:10	d < 1:10	0,09	0,09	0,09	6,44	0,12	0,14	0,13	8,86	0,60	0,58	0,59	44,53
**MR 416 /15 SR**	Guidimaka	Mamayel	n.s.	cap	f	3	**d 1:960**	**d 1:160**	d < 1:10	0,61	0,45	0,53	37,87	0,75	0,68	0,72	48,53	0,85	0,86	0,86	65,04
**MR 417 /15 SR**	Guidimaka	Mamayel	n.s.	cap	f	2	**d 1:480**	**d 1:80**	d < 1:10	0,50	0,46	0,48	34,63	0,48	0,48	0,48	32,49	0,31	0,31	0,31	23,37
**MR 418 /15 SR**	Guidimaka	Mamayel	n.s.	cap	f	2	**d 1:120**	**d 1:100**	d < 1:10	0,21	0,16	0,18	13,17	0,62	0,41	0,52	35,06	0,55	0,51	0,53	40,08
**MR 419 /15 SR**	Guidimaka	Mamayel	n.s.	cap	f	3	d < 1:10	d < 1:10	d < 1:10	0,22	0,24	0,23	16,44	0,16	0,16	0,16	10,90	0,28	0,31	0,29	22,27
**MR 420 /15 SR**	Guidimaka	Mamayel	n.s.	cap	f	4	**d 1:60**	**d 1:60**	d < 1:10	0,69	0,56	0,62	44,58	0,64	0,72	0,68	45,72	0,36	0,38	0,37	27,85
**MR 421 /15 SR**	Guidimaka	Mamayel	n.s.	cap	f	3	d < 1:10	d < 1:10	d < 1:10	0,11	0,10	0,10	7,48	0,12	0,14	0,13	8,74	0,20	0,22	0,21	15,86
**MR 422 /15 SR**	Guidimaka	Mamayel	n.s.	cap	f	3	d < 1:10	d < 1:10	d < 1:10	0,23	0,15	0,19	13,72	0,23	0,22	0,23	15,32	0,46	0,48	0,47	35,61
**MR 423 /15 SR**	Guidimaka	Mamayel	n.s.	cap	f	3	d < 1:10	d < 1:10	d < 1:10	0,11	0,09	0,10	7,13	0,22	0,25	0,23	15,86	0,63	0,66	0,65	49,15
**MR 424 /15 SR**	Guidimaka	Mamayel	n.s.	cap	f	2	**d 1:80**	**d 1:480**	d < 1:10	1,87	0,96	1,42	101,37	1,14	1,66	1,40	94,55	0,26	0,28	0,27	20,76
**MR 425 /15 SR**	Guidimaka	Mamayel	n.s.	cap	f	4	**d 1:120**	**d 1:80**	d < 1:10	0,21	0,15	0,18	12,59	0,29	0,30	0,30	20,14	0,39	0,40	0,39	29,68
**MR 426 /15 SR**	Guidimaka	Mamayel	n.s.	cap	f	2	**d 1:60**	**d 1:20**	d < 1:10	0,15	0,15	0,15	20,51	0,17	0,17	0,17	13,52	0,38	0,49	0,44	33,13
**MR 427 /15 SR**	Guidimaka	Mawayel	n.s.	cap	f	3	**d 1:320**	**d 1:60**	**d 1:15**	1,64	1,19	1,42	101,28	1,22	1,49	1,35	91,45	0,64	0,64	0,64	50,57
**MR 428 /15 SR**	Guidimaka	Mawayel	n.s.	cap	f	2	d < 1:10	d < 1:10	d < 1:10	0,13	0,10	0,12	8,25	0,34	0,28	0,31	20,98	1,94	1,91	1,93	152,22
**MR 429 /15 SR**	Guidimaka	Mawayel	n.s.	cap	f	3	d < 1:10	d < 1:10	d < 1:10	0,17	0,21	0,19	13,65	0,36	0,37	0,36	24,65	0,67	0,68	0,68	53,39
**MR 430 /15 SR**	Guidimaka	Mawayel	n.s.	cap	f	1	d < 1:10	d < 1:10	d < 1:10	0,14	0,13	0,14	9,78	0,36	0,32	0,34	22,73	0,87	0,86	0,87	68,64
**MR 431 /15 SR**	Guidimaka	Mawayel	n.s.	cap	f	3	**d 1:1280**	**d 1:110**	**d 1:30**	0,57	0,55	0,56	77,65	0,77	0,76	0,77	101,97	2,67	2,61	2,64	208,44
**MR 432 /15 SR**	Guidimaka	Mawayel	n.s.	cap	f	2	**d 1:160**	**d 1:100**	d < 1:10	0,82	0,52	0,67	47,94	1,33	1,33	1,33	89,72	0,34	0,34	0,34	26,97
**MR 433 /15 SR**	Guidimaka	Mawayel	n.s.	cap	f	2	**d 1:320**	**d 1:80**	d < 1:10	0,42	0,29	0,36	25,54	0,97	0,76	0,87	58,69	0,35	0,35	0,35	27,78
**MR 434 /15 SR**	Guidimaka	Mawayel	n.s.	cap	f	3	**d 1:240**	**d 1:60**	**d 1:30**	0,28	0,20	0,24	17,07	0,45	0,43	0,44	29,97	0,70	0,72	0,71	56,33
**MR 435 /15 SR**	Guidimaka	Mawayel	n.s.	cap	f	4	d < 1:10	d < 1:10	d < 1:10	0,12	0,11	0,12	8,26	0,22	0,23	0,23	15,43	0,53	0,53	0,53	41,61
**MR 436 /15 SR**	Guidimaka	Mawayel	n.s.	cap	f	3	**d 1:160**	**d 1:90**	d < 1:10	1,18	0,86	1,02	73,22	1,88	1,53	1,70	115,17	0,82	0,82	0,82	65,12
**MR 437 /15 SR**	Guidimaka	Mawayel	n.s.	cap	f	3	**d 1:80**	**d 1:80**	d < 1:10	0,24	0,25	0,24	17,45	0,34	0,39	0,36	24,48	0,39	0,38	0,39	30,47
**MR 438 /15 SR**	Guidimaka	Mawayel	n.s.	cap	f	3	d < 1:10	d < 1:10	d < 1:10	0,11	0,10	0,10	7,20	0,40	0,24	0,32	21,56	2,75	2,76	2,75	217,71
**MR 439 /15 SR**	Guidimaka	Mawayel	n.s.	cap	f	4	d < 1:10	d < 1:10	d < 1:10	0,15	0,12	0,14	9,82	0,29	0,30	0,29	19,80	0,46	0,47	0,47	36,87
**MR 440 /15 SR**	Guidimaka	Mawayel	n.s.	cap	f	4	**d 1:80**	**d 1:480**	**d 1:30**	0,32	0,18	0,25	18,19	0,41	0,51	0,46	30,92	0,50	0,48	0,49	38,76
**MR 441 /15 SR**	Guidimaka	Mawayel	n.s.	cap	f	2	**d 1:160**	**d 1:40**	d < 1:10	0,93	0,38	0,65	46,84	1,73	1,91	1,82	123,09	2,36	2,34	2,35	185,97
**MR 442 /15 SR**	Guidimaka	Mawayel	n.s.	ov	f	3	d < 1:10	d < 1:10	d < 1:10	0,19	0,19	0,19	13,51	0,19	0,21	0,20	13,60	0,77	0,78	0,78	61,54
**MR 443 /15 SR**	Guidimaka	Mawayel	n.s.	ov	f	2	**d 1:15**	d < 1:10	d < 1:10	0,16	0,14	0,15	10,72	0,34	0,30	0,32	21,69	0,55	0,55	0,55	43,35
**MR 444 /15 SR**	Guidimaka	Mawayel	n.s.	ov	f	3	**d 1:40**	**d 1:30**	d < 1:10	0,12	0,13	0,13	9,15	0,30	0,25	0,27	18,59	0,27	0,27	0,27	21,24
**MR 445 /15 SR**	Guidimaka	Mawayel	n.s.	ov	f	2	**d 1:60**	**d 1:120**	d < 1:10	1,61	1,14	1,38	98,52	1,72	1,92	1,82	123,28	0,51	0,52	0,51	40,38
**MR 446 /15 SR**	Guidimaka	Mawayel	n.s.	ov	m	3	**d 1:480**	**d 1:100**	d < 1:10	0,30	0,27	0,28	20,33	0,34	0,38	0,36	24,48	0,57	0,59	0,58	45,84
**MR 447 /15 SR**	Guidimaka	Mawayel	n.s.	ov	f	2	d < 1:10	d < 1:10	d < 1:10	0,09	0,09	0,09	12,37	0,12	0,13	0,12	10,15	0,78	0,81	0,80	63,15
**MR 448 /15 SR**	Guidimaka	Mawayel	n.s.	ov	f	3	**d 1:120**	**d 1:120**	d < 1:10	0,55	0,51	0,53	37,85	0,51	0,60	0,55	37,50	0,29	0,29	0,29	22,90
**MR 449 /15 SR**	Guidimaka	Mawayel	n.s.	ov	f	4	**d 1:640**	**d 1:120**	**d 1:30**	0,71	0,47	0,59	42,15	0,63	0,72	0,68	45,69	0,81	0,81	0,81	63,91
**MR 450 /15 SR**	Guidimaka	Mawayel	n.s.	ov	m	2	d < 1:10	d < 1:10	d < 1:10	0,20	0,23	0,21	15,13	0,34	0,43	0,39	26,20	0,63	0,64	0,64	50,41
**MR 451 /15 SR**	Guidimaka	Mawayel	n.s.	ov	f	3	d < 1:10	d < 1:10	d < 1:10	0,14	0,14	0,14	19,47	0,21	0,20	0,21	16,79	1,27	1,27	1,27	100,47
**MR 452 /15 SR**	Guidimaka	Mawayel	n.s.	ov	f	3	d < 1:10	d < 1:10	d < 1:10	0,08	0,07	0,07	10,34	0,08	0,08	0,08	6,79	0,10	0,10	0,10	7,71
**MR 453 /15 SR**	Guidimaka	Mawayel	n.s.	ov	f	4	**d 1:640**	**d 1:160**	d < 1:10	1,82	1,51	1,66	119,11	1,39	1,40	1,40	94,59	0,70	0,71	0,71	55,72
**MR 454 /15 SR**	Guidimaka	Mawayel	n.s.	ov	f	4	**d 1:60**	**d 1:30**	d < 1:10	0,76	0,78	0,77	106,65	0,84	0,87	0,85	69,40	0,12	0,12	0,12	9,30
**MR 455 /15 SR**	Guidimaka	Mawayel	n.s.	ov	f	4	**d 1:640**	**d 1:60**	**d 1:20**	1,01	0,89	0,95	67,82	0,98	1,09	1,03	69,95	0,72	0,73	0,73	57,32
**MR 456 /15 SR**	Guidimaka	Mawayel	n.s.	ov	f	2	**d 1:60**	**d 1:40**	**d 1:15**	1,63	1,40	1,51	108,34	1,65	1,94	1,80	121,66	0,59	0,60	0,59	46,85
**MR 457 /15 SR**	Guidimaka	Mawayel	n.s.	ov	f	2	d < 1:10	d < 1:10	d < 1:10	0,09	0,09	0,09	6,40	0,12	0,13	0,13	8,66	0,48	0,48	0,48	37,86

**MR 458 /15 SR**	Guidimaka	Hel Oboye	n.s.	cap	f	2	d < 1:10	d < 1:10	d < 1:10	0,11	0,09	0,10	7,24	0,15	0,17	0,16	10,85	0,48	0,51	0,49	39,11
**MR 459 /15 SR**	Guidimaka	Hel Oboye	n.s.	cap	f	2	**d 1:160**	**d 1:80**	**d 1:15**	0,50	0,31	0,41	28,98	0,61	0,63	0,62	41,91	0,82	0,92	0,87	68,59
**MR 460 /15 SR**	Guidimaka	Hel Oboye	n.s.	cap	f	2	**d 1:2560**	**d 1:640**	**d 1:15**	2,59	1,64	2,11	151,08	2,01	2,06	2,04	137,90	2,21	2,34	2,28	180,07
**MR 461 /15 SR**	Guidimaka	Hel Oboye	n.s.	cap	f	1	d < 1:10	d < 1:10	d < 1:10	0,11	0,23	0,17	12,10	0,16	0,36	0,26	17,59	0,52	0,57	0,55	43,08
**MR 462 /15 SR**	Guidimaka	Hel Oboye	n.s.	cap	f	2	**d 1:2560**	**d 1:640**	d < 1:10	2,41	2,00	2,20	157,69	2,02	2,08	2,05	138,54	2,00	2,10	2,05	162,00
**MR 463 /15 SR**	Guidimaka	Hel Oboye	n.s.	cap	f	1	**d 1:480**	**d 1:60**	d < 1:10	1,45	0,81	1,13	80,56	1,63	1,70	1,66	112,59	0,70	0,72	0,71	56,11
**MR 464 /15 SR**	Guidimaka	Hel Oboye	n.s.	cap	f	1	**d 1:80**	**d 1:80**	**d 1:30**	2,12	1,55	1,83	131,13	1,93	2,13	2,03	137,17	0,74	0,79	0,76	60,46
**MR 465 /15 SR**	Guidimaka	Hel Oboye	n.s.	cap	f	1	**d 1:1920**	**d 1:480**	**d 1:20**	1,51	0,88	1,19	85,45	1,71	1,63	1,67	112,94	1,27	1,39	1,33	104,93
**MR 466 /15 SR**	Guidimaka	Hel Oboye	n.s.	cap	f	1	**d 1:480**	**d 1:240**	**d 1:40**	2,38	1,60	1,99	142,05	1,93	2,07	2,00	135,38	0,63	0,75	0,69	54,56
**MR 467 /15 SR**	Guidimaka	Hel Oboye	n.s.	cap	m	1	**d 1:80**	**d 1:60**	d < 1:10	1,20	0,81	1,00	71,80	1,35	1,50	1,42	96,33	1,00	0,98	0,99	78,90
**MR 468 /15 SR**	Guidimaka	Hel Oboye	n.s.	ov	f	1	**d 1:480**	**d 1:160**	d < 1:10	1,54	0,83	1,18	84,61	1,68	1,63	1,66	112,13	0,78	0,79	0,78	62,32
**MR 469 /15 SR**	Guidimaka	Hel Oboye	n.s.	ov	f	1	**d 1:1280**	**d 1:240**	d 1:15	1,68	0,94	1,31	93,60	1,82	1,71	1,77	119,57	1,30	1,28	1,29	102,75
**MR 470 /15 SR**	Guidimaka	Hel Oboye	n.s.	ov	f	2	**d 1:80**	**d ≥ 1:60**	d 1:20	0,37	0,35	0,36	50,05	0,50	0,48	0,49	40,03	0,89	0,90	0,90	71,62
**MR 471 /15 SR**	Brakna	Tachott	n.s.	ov	f	4	**d 1:80**	**d 1:40**	d < 1:10	0,14	0,14	0,14	19,53	0,26	0,25	0,26	20,77	0,37	0,38	0,37	29,88
**MR 472 /15 SR**	Brakna	Tachott	n.s.	ov	f	5	**d 1:160**	**d 1:40**	d < 1:10	1,38	0,39	0,88	63,19	1,76	1,67	1,72	116,23	1,84	1,81	1,83	145,58
**MR 473 /15 SR**	Brakna	Tachott	n.s.	ov	f	4	d < 1:10	d < 1:10	d < 1:10	0,10	0,11	0,11	14,72	0,19	0,19	0,19	15,75	0,82	0,91	0,87	68,98
**MR 474 /15 SR**	Brakna	Tachott	n.s.	ov	f	5	**d 1:60**	**d 1:40**	d 1:10	0,64	0,35	0,49	35,26	0,82	0,90	0,86	58,09	0,98	0,99	0,99	77,43
**MR 475 /15 SR**	Brakna	Tachott	n.s.	cap	f	4	d < 1:10	d < 1:10	d < 1:10	0,14	0,13	0,13	9,64	0,60	0,85	0,72	48,83	1,20	1,21	1,21	94,92
**MR 476 /15 SR**	Brakna	Tachott	n.s.	cap	f	3	**d ≥ 1:120**	**d ≥ 1:60**	d < 1:10	1,25	1,18	1,22	168,42	1,89	1,89	1,89	153,45	1,24	1,37	1,30	102,41
**MR 477 /15 SR**	Brakna	Tachott	n.s.	ov	f	5	d < 1:10	d < 1:10	d < 1:10	0,09	0,09	0,09	13,12	0,12	0,12	0,12	9,78	0,33	0,32	0,32	25,55
**MR 479 /15 SR**	Brakna	Tachott	n.s.	ov	f	3	**d 1:60**	**d 1:20**	d 1:10	0,15	0,15	0,15	20,54	0,16	0,16	0,16	13,30	0,67	0,62	0,64	50,61
**MR 481 /15 SR**	Brakna	Tachott	n.s.	cap	f	5	d < 1:10	d < 1:10	d < 1:10	0,12	0,11	0,11	8,12	0,24	0,23	0,24	16,00	0,34	0,34	0,34	26,51
**MR 482 /15 SR**	Brakna	Tachott	n.s.	cap	f	5	**d 1:320**	**d 1:80**	d 1:10	1,50	0,74	1,12	80,06	1,82	1,69	1,75	118,69	0,57	0,61	0,59	47,04
**MR 483 /15 SR**	Brakna	Tachott	n.s.	ov	m	4	d < 1:10	d < 1:10	d < 1:10	0,09	0,08	0,08	11,70	0,10	0,10	0,10	8,22	0,18	0,17	0,18	14,05
**MR 486 /15 SR**	Brakna	Tachott	n.s.	ov	f	3	**d 1:240**	**d 1:60**	d < 1:10	0,23	0,25	0,24	17,29	0,44	0,48	0,46	31,15	0,49	0,50	0,50	38,90
**MR 489 /15 SR**	Brakna	Tachott	n.s.	cap	f	5	**d 1:960**	**d 1:160**	d < 1:10	1,50	1,46	1,48	105,99	1,47	1,51	1,49	100,69	0,88	0,93	0,90	71,07
**MR 494 /15 SR**	Brakna	Tachott	n.s.	cap	f	6	**d 1:960**	**d 1:320**	**d 1:30**	1,86	1,62	1,74	124,53	1,61	1,77	1,69	114,37	2,93	2,96	2,94	231,31
**MR 497 /15 SR**	Gharbi	n.s.	n.s.	s.r.	n.s.	n.s.	d < 1:10	d < 1:10	d < 1:10	0,10	0,09	0,10	7,07	0,14	0,23	0,19	12,65	0,27	0,27	0,27	21,20
**MR 498 /15 SR**	Gharbi	n.s.	n.s.	s.r.	n.s.	n.s.	**d 1:240**	**d 1:60**	d < 1:10	0,25	0,18	0,22	15,42	0,63	0,55	0,59	39,76	0,50	0,52	0,51	39,85
**MR 500 /15 SR**	Gharbi	n.s.	n.s.	s.r.	n.s.	n.s.	**d 1:30**	**d 1:160**	d < 1:10	0,21	0,24	0,22	15,97	0,35	0,30	0,33	21,99	1,65	1,65	1,65	129,68

n.s.: not specified
s.r.: small ruminants
SNT: Serum neutralisation test
